# Rotavirus replication is correlated with S/G2 interphase arrest of the host cell cycle

**DOI:** 10.1371/journal.pone.0179607

**Published:** 2017-06-16

**Authors:** Selene Glück, Antonino Buttafuoco, Anita F. Meier, Francesca Arnoldi, Bernd Vogt, Elisabeth M. Schraner, Mathias Ackermann, Catherine Eichwald

**Affiliations:** 1 Institute of Virology, University of Zurich, Zurich, Switzerland; 2 Department of Medicine, Surgery and Health Sciences, University of Trieste, Trieste, Italy; 3 Institute of Anatomy, Vetsuisse, University of Zurich, Zurich, Switzerland; Institute of Human Virology, UNITED STATES

## Abstract

In infected cells rotavirus (RV) replicates in viroplasms, cytosolic structures that require a stabilized microtubule (MT) network for their assembly, maintenance of the structure and perinuclear localization. Therefore, we hypothesized that RV could interfere with the MT-breakdown that takes place in mitosis during cell division. Using synchronized RV-permissive cells, we show that RV infection arrests the cell cycle in S/G2 phase, thus favoring replication by improving viroplasms formation, viral protein translation, and viral assembly. The arrest in S/G2 phase is independent of the host or viral strain and relies on active RV replication. RV infection causes cyclin B1 down-regulation, consistent with blocking entry into mitosis. With the aid of chemical inhibitors, the cytoskeleton network was linked to specific signaling pathways of the RV-induced cell cycle arrest. We found that upon RV infection Eg5 kinesin was delocalized from the pericentriolar region to the viroplasms. We used a MA104-Fucci system to identify three RV proteins (NSP3, NSP5, and VP2) involved in cell cycle arrest in the S-phase. Our data indicate that there is a strong correlation between the cell cycle arrest and RV replication.

## Introduction

Rotavirus (RV), a member of the *Reoviridae* family, is an icosahedral, non-enveloped, triple-layered particle responsible for severe diarrhea and dehydration in infants and young animals. During the RV replication cycle, receptor-mediated endocytosis and partial digestion of the infectious particle lead to the release of double-layered particles (DLPs) to the cytosol, which are transcriptionally active and initiate replication of the viral progeny [1, 2]. As a consequence of infection some initial events have been identified: i) shut-off of the host translation machinery through a mechanism involving NSP3 [3–7], ii) blockage of the innate immune response, involving NSP1 and VP3 by mechanisms that are viral strain dependent [5, 8], and iii) formation of viroplasms, cytoplasmic structures that serve as viral factories of newly made viral particles. Viroplasms contain four viral structural proteins (VP1, VP2, VP3, and VP6), three viral nonstructural proteins (NSP2, NSP5, and NSP6), viral-derived +ssRNA and dsRNA as well as host components, not yet fully characterized [9–11]. An additional nonstructural protein, NSP4, was found associated to viroplasms [12]. Within viroplasms replication of +ssRNA and packaging of the viral dsRNA genome segments in viral cores is followed by the acquisition of a layer of trimeric VP6 to form DLPs. Once assembled, competent DLPs bud into the ER to acquire the third layer composed of glycoprotein VP7 and the spike protein VP4 [13–15]. Viroplasms are highly dynamic structures able to coalesce between them, changing from small structures at early time points post-infection to large ones at later time points [15–17]. The dynamics of the viroplasms involve their movement from the cell periphery towards the perinuclear region. Both coalescence and perinuclear distribution of the viroplasms depend on a stable MT-network [18]. It has been observed that viroplasms are encaged by acetylated-MTs, which provide a stabilized environment for the formation and maintenance of the viroplasm-structure. Additionally, the kinesin motor of the Eg5 family has also been implicated in the viroplasm dynamics permitting its perinuclear localization and structural support [18]. The viral proteins NSP5, VP2, and NSP2 are directly involved in the formation of viroplasms and act as recruiters for the components of the viral replication intermediates [19–22]. Accordingly, NSP2 and VP2 have different tasks in the viroplasm dynamics: NSP2 associate to MTs permitting viroplasms coalescence, whereas VP2 has a role in viroplasms perinuclear distribution [18, 23]. On the other side, NSP2 and VP2 also regulate NSP5 hyperphosphorylation by triggering phosphorylation of Ser-67 in NSP5 by casein kinase 1 alpha. The exact role of NSP5 in the RV replicative cycle remains yet to be elucidated [24, 25].

The cell cycle is a series of consecutive biochemical switches allowing the host genome replication at the S-phase of the interphase and subsequently to be equally divided during mitosis, generating two identical daughter cells [26]. Between S-phase and mitosis the so-called gap phases, G1 and G2, permit the cell to grow and prepare for the subsequent stages. Upon harsh conditions, such as the absence of mitogens, the cell arrest at specific restriction points and stay in the resting phase (G0). Cyclin-dependent kinases (CDKs) are key components for the entry into the cell cycle phases and are only active when bound to cyclins. Specific cyclins accumulate at the beginning of a new phase, having the highest expression level once the phase is established. In contrast, the concentration of CDKs remains stable during the whole cell cycle. In eukaryotes, five different CDKs have been identified, which can be found in their active forms in phases G1 (CDK4 and CDK6), S (CDK2), G2 and mitosis (CDK1). Also, CDK7 acts with cyclin H as a CDK-activating kinase (CAK) [27, 28]. The control of the cell cycle depends on post-translational modifications (PTMs) of cyclins (e.g. by phosphorylation), which lead to their activation or deactivation. In particular, poly-ubiquitination by the ubiquitin ligase APC/C (anaphase-promoting complex), which leads to the 26S proteasome-mediated degradation, is responsible for cyclin deactivation [29]. Entry into mitosis relies on an increase in the concentration of cyclin B1/CDK1 complexes by the rise of cyclin B1 during G2-phase and its accumulation during mitosis. The active cyclin B1/CDK1 complex localizes at the centrosome [30]. Also, the entry in mitosis is associated with the phosphorylation of downstream helper kinases, cytoskeletal proteins such as Eg5, chromosomal proteins, and lamins, as well as with nuclear envelope breakdown. Interestingly, Eg5 is an MT motor protein responsible for the centrosome separation and bipolar spindle assembly during mitosis permitting the movement towards the plus end of MTs and establishing the antiparallel array of MTs spindles. When cells enter mitosis, CDK1 phosphorylates Eg5 in the nucleus, resulting in a stronger binding association to MTs [31]. During the interphase, Eg5 presumably binds to ribosomes via MTs and enhances translation efficiency [32]. The regulation of MT polymerization and depolymerization is dependent on MT-associated proteins (MAPs) (33) and PTMs (34, 35). The MTs in the interphase are longer and much more stable than those in mitosis [33]. At the onset of mitosis, the interphase MT-network converts into short and highly dynamic MTs surrounding each of the centrosomes, where the MT-nucleation increases, resulting in dense and dynamic arrays of MT-spindle fibers. Also, the molecular motors and MAPs, contribute to the generation of a bipolar array of MTs and favor the local increase of the MT-nucleation in the centrosomes surface [34–38].

How RV avoids the MT-network breakdown to permit its replication remains an important question. We show that the RV-induced cell cycle arrest is dependent on viral replication. By using a set of host cell inhibitors, we show that actin and MT-networks, as well as Eg5 motor protein pathways, are implicated in the cell cycle arrest. RV subverts Eg5, delocalizing it towards the viroplasms together with its phosphorylation during the interphase. Finally, using Fucci sensors in RV-permissive MA104 cells, we show that NSP3, NSP5, and VP2 can arrest cells in S-phase.

## Materials and methods

### Cells and viruses

MA104 (embryonic rhesus monkey kidney, ATCC®CRL-2378), MA104-Fucci, CV-1 (African green monkey kidney fibroblast, generously donated by Max L. Nibert, Harvard Medical School), MDCK (Madin-Darby canine kidney, NBL-2, ATCC®CCL-34), ATR-U20S Fucci (Human bone osteosarcoma epithelial cells, A. Meier, unpublished data) hereafter named as U2OS cells and HEK293T (embryonic human kidney epithelial, ATCC®CRL-3216) cells were cultured in Dulbecco’s modified Eagle’s media (DMEM, Gibco®BRL) supplemented with 10% fetal calf serum (FCS)(AMIMED, Bioconcept, Switzerland). Caco-2 (colorectal adenocarcinoma human intestinal epithelial cell line, ATCC®HTB-37) cells were cultured in DMEM and supplemented with 20% FCS. NSP5-EGFP/MA104 cell line [16] was cultured in DMEM supplemented with 10% FCS and 800 μg/ml geneticin (Sigma-Aldrich). All the cultures were kept routinely in penicillin (100 U/ml) and streptomycin (100 μg/ml). Simian RV strain SA11 (G3P6[1]) [39], rhesus RV strain RRV (G3P5B[3]) [40] and porcine RV strain OSU (G5P9[7]) [41] were propagated, grown and purified as described by Arnold *et al*., 2009[42]. RV titers were measured in Viroplasm Forming Units per milliliter (VFU/ml) using NSP5-EGFP/MA104 cell line, as described in detail by Eichwald *et al*., 2012 [18]. In this cell line, upon RV infection NSP5-EGFP gets recruited in the newly formed viroplasms. We found that a MOI of 25 VFU/cell is the minimal amount of viral particles necessary for the infection of the complete cell monolayer.

### Antibodies and reagents

A full list of the antibodies used in this study is provided in S1 Table. Thymidine, propidium iodide, psoralen, nocodazole, monastrol, ciliobrevinD, MG132, cycloheximide, tubacin and sodium butyrate were purchased from Sigma-Aldrich (St Louis, MO, USA). Cytochalasin B was purchased from AppliChem GmbH (Darmstadt, Germany). Lactacystin and purvalanol were purchased from Santa Cruz Biotechnology, Inc. UBEI-41 was acquired from Biogenova, USA.

### Plasmid constructions

The lentiviral plasmids pFUG-Fucci-G1o and pFUG-Fucci-SG2Mg were obtained by PCR amplification of pFucci-G1 Orange (cat# AM-V9001, Amalgaam, MBL, Ltd.) and pFucci-S/G2/M Green (cat# AM-V9014, Amalgaam, MBL, Ltd.) using specific primers to insert BamHI and EcoRI restriction sites and subsequently, ligated in pFUGW [43] between BamHI and EcoRI restriction sites. The plasmids pCMVΔR8.2 and pMD2G(VSV-G) have been described previously [44].

The plasmids pCI-CFP-VP6, pCI-CFP-NSP1, and pCI-CFP-NSP4 were obtained by PCR amplification of pENTR-VP6 strain EC (G3P[16]) [45], pUC19-NSP1strain SA11 and pHSV-NSP4-EGFP from strain WA (G1P[8]) (kindly provided by Dr. Andrea Laimbacher, University of Zurich, Zurich, Switzerland) using specific primers to insert MluI and NotI restriction sites and then ligated in frame in pCI-CFP-N between MluI and NotI. The plasmid pCI-CFP-N and pCI-atg-CFP were obtained by PCR amplification of pscAAV-eCFP (kindly provided by Prof. C. Fraefel, University of Zurich, Zurich, Switzerland) with specific primers to insert XhoI and MluI restriction sites, followed by ligation in pCI-neo (Promega) between XhoI and MluI. The NSP1 gene was cloned from SA11 4F (G3 P[6])-infected cells. The cDNA was obtained by reverse transcription, using random hexamers (IDT) and Moloney Murine Leukemia Virus Reverse Transcriptase (Life Technologies). Subsequently, the cDNA spanning the whole genome segment 5 was amplified by PCR using specific primers and cloned in pUC19 (a gift from Joachim Messing, (Addgene plasmid #50005) [46]) using the SmaI restriction site.

The plasmids pCI-VP4-CFP and pCI-NSP3-CFP were obtained by PCR amplification of pCMV-VP4 strain SA11 [47] and pcDNA-NSP3 strain SA11 [48] with specific primers to insert XhoI and MluI, respectively and subsequently ligated in pCI-C-CFP(M/N) between XhoI/MluI restriction sites. The plasmid pCI-C-CFP (M/N) was obtained by PCR amplification of pscAAV-eCFP with specific primers to insert MluI and NotI restriction sites and ligated in pCI-neo (Promega) between MluI/NotI. The plasmids pCI-NSP2-CFP, pCI-VP2-CFP, pCI-NSP5-CFP, pCI-NSP5(S67A)-CFP, and pCI-NSP5(S67D)-CFP were obtained by digestion of the plasmid pCI-C-CFP(M/N) with MluI and NotI restriction enzymes to obtain the fragment CFP and then ligated into pCI-NSP2, pCI-VP2, pCI-NSP5, pCI-NSP5(S67A), and pCI-NSP5(S67D) between MluI/NotI restriction sites. The plasmids pCI-NSP2, pCI-VP2, pCI-NSP5, pCI-NSP5(S67A), and pCI-NSP5(S67D) were obtained by PCR amplification of pcDNA-NSP2 (strain SA11) [16], pcDNA-VP2 (strain SA11) [21], pcDNA-NSP5 (strain OSU) [16], pT_7_v-NSP5/S67A [24] and pT_7_V-NSP5(S63, 65A/S67D) [24] using specific primers to insert EcoRI and MluI restriction sites, respectively and then ligated in pCI-neo (Promega) between EcoRI/MluI. All the oligonucleotides were obtained from Microsynth AG, Switzerland and are listed in S2 Table.

### Preparation of MA104-Fucci cells

The Fucci cell cycle sensor consists of a fluorescent protein-based system that employs both red (mKO2) and green (mAG) fluorescent protein fused to the cell cycle regulators Cdt1 (Cdt1-mKO2; red) and geminin (geminin-mAG; green), respectively [49]. Lentiviral particles carrying Fucci-G1o (LV Fucci-G1o) or Fucci-S/G2/Mg (LV Fucci-S/G2/Mg) were prepared by transfection of a 100 mm tissue culture Petri dish at 70% confluence of HEK293T cells. The cell medium was exchanged 4 hours before transfection by DMEM supplemented with 10% FCS and penicillin/streptomycin. Cells were transfected with 15 μg pCMVR8.9, 10 μg pVSVg and 20 μg pFUG-Fucci-G1o (for LV Fucci-G1o) or pFUG-Fucci-S/G2/Mg (for LV Fucci- S/G2/Mg) diluted in 1 ml of DMEM containing 40μl PEI (1 mg/ml) (polyethyleneimine, linear, MW 25000; Polysciences, Inc. USA) and incubated for 10 min at room temperature. The transfection mix was added dropwise to the cells. At 72 hpt (hours post-transfection), the medium was harvested, centrifuged at 3,000 x g for 10 min and filtered through a 0.45μm membrane filter. For lentiviral transduction, 30% confluent MA104 cells in a 25-cm2 tissue culture flask were incubated for 1 hour at 37°C in media containing 1ml of LV-Fucci G1o, 1 ml LV-Fucci-S/G2/Mg and 8 μg/ml polybrene (Hexadimethrine bromide, Sigma). Then, 3 ml of DMEM supplemented with 10% FCS were added and incubated at 37°C and 5% CO2 for seven days. Two 75-cm2 tissue culture bottles containing 30–50% confluence of transduced cells were sorted selecting for positive red and green fluorescing cells using a BD FACSAria III cell sorter using BD FACSDiva Software. The isolated MA104-Fucci cells were seeded and propagated as described for wild-type MA104 cells.

### Cell synchronization by double thymidine block

Cells were synchronized at the beginning of G1/S-phase as previously described [50]. Briefly, 40% confluent cells in 100 mm tissue culture dishes were incubated for 12h in the presence of 3 mM thymidine (Sigma-Aldrich). The cells were released for 10h by washing out the thymidine and seeding the cells at 50% confluence in 60 mm tissue culture dishes. The cells were then blocked again in 3 mM thymidine for 12h. Cells were released from thymidine block by washing three times with phosphate buffer saline (PBS) [1mM KH_2_PO_4_, 3mM Na_2_HPO_4_•7H_2_O, 155 mM NaCl, pH 7.4]. For infection with RV, cells were incubated for 1h at 4°C followed by incubation for the indicated time post-release at 37°C and 5% CO2.

### Cell cycle analysis by DNA content

After trypsinization, 1x10^6^ cells were centrifuged for 5 min at 530 x g. The cellular pellet was fixed with ice-cold 70% ethanol and incubated for 16 h at -20°C. The cells were centrifuged at 530 x g at 4°C for 5 min, washed once with PBS and stained in the dark for 40 min at 37°C with a 500μl propidium iodide (PI) solution (0.05% Triton X-100, 5 U/ml RNaseA and 50μg/ml propidium iodide in PBS). Afterward, cells were centrifuged for 5 min at 530 x g, resuspended in 1 ml PBS, filtered using a cell strainer snap-cap tube (BD Falcon™) and immediately acquired in a Gallios™ Flow Cytometer (Beckman Coulter, Inc). For this purpose, 25’000 events were acquired, exciting at 488 nm with an argon laser and filter band of 695/40 nm. Data were analyzed for cell cycle using the cell cycle algorithm of FlowJo V.10.0.8r1 software (FlowJo.LLC). The cell cycle curves for DNA content were obtained using Watson or Dean-Jett-Fox (DJF) mathematical model [51]. The G1 and G2 peaks were constrained using the peaks, in each experimental set, of non-infected cells at late time post-release. The relative (S+G2)/G1 ratio was obtained by considering as value of 1 the non-infected cells at 0 hpr or CFP alone expressing cells.

The following empiric formula was developed to integrate the plots for DNA content obtained from the cell cycle analysis when cells were RV-infected (RV), treated only with a chemical compound (D) or both (RV+D), to fit with the observed biological behavior. The formula is expressed as a ratio from RV-infected cells and only drug treated cells with respect to RV-infected and drug treated cells. A log_10_ of the ratio was applied to obtain negative (<1) or positive (>1) values when non-linked or linked to the RV induced cell cycle arrest with the physiological pathway, respectively.

$$\log_{10}\;ratio=\log_{10}\;\left[\left(\sqrt[{2}]{\left(\frac{\%S}{\%G2})\text{RV}\times (\frac{\%S}{\%G2}\right)}D\right)/\left(\frac{\%S}{\%G2}\right)RV+D\right]$$

Herein, the %S and the % G2 are the percentage of cells in S and G2 phases, respectively. The RV, D, and RV+D correspond to the data obtained from RV-infected cells, drug-treated cells and RV-infected and drug-treated cells, respectively. The formula was designed so that the value obtained can discriminate the following conditions: (i) the drug intrinsically arrests cells in S-phase, (ii) the drug cannot interfere with RV-arrest and (iii) the drug prevents the RV-arrest in S-phase. According to the log_10_ ratio, the conditions i) and ii) will provide a value <0 and the condition iii) will provide a value>0.

### Cell cycle analysis by Fucci sensors

MA104-Fucci cells were harvested after trypsin treatment and centrifuged for 2 min at 530 x g. The cellular pellet was resuspended in 1ml of DMEM supplemented with 10% FCS and filtered using a cell strainer snap-cap tube (BD Falcon™) and immediately acquired in a Gallios™ Flow Cytometer (Beckman Coulter, Inc). To this end, a total of 50’000 events were acquired exciting with a 488 nm and 561 nm lasers followed by default filters of 525/50nm (mAG, green) and 575/60 nm (mKO2, orange), respectively. Additionally, when acquiring CFP-transfecting cells, the events were also excited with a 405 nm laser followed by a filter default of 550/40 nm.

### Transfection of MA104-Fucci cells

4x10^5^ cells in a 60 mm2 tissue culture Petri dish were transfected with 2μg of DNA plasmid, 9μl Lipofectamine® 2000 transfection reagent (ThermoFisher, Scientific) diluted in 600 μl Opti-MEM (ThermoFisher, Scientific) following the manufacturer instructions. At 5 hpt, the transfection medium was replaced with DMEM supplemented with 10%FCS and penicillin/streptomycin. Cells were harvested at 24 hpt.

### RV inactivation

The virus was inactivated as described by Groene and Shaw [52] with modifications. Purified RV [42] was distributed in aliquots of 50μl in 96-well multiwell plates and mixed with 40μg/ml of psoralen (AMT, 4’-aminomethyl trioxsalen hydrochloride). The plate was wrapped in aluminum foil and incubated on ice for 15min. Then, the plate was UV-crosslinked with 960 mJ/cm3 for 30 min (UVC500, Hoefer).

### RV particles negative staining

The viral particles were adsorbed for 10 min on carbon-coated Parlodion films mounted on 300 meshes per inch copper grids (Electron Microscopy Sciences, Hatfield, PA, USA). Samples were washed once with distilled water and stained with saturated uranyl acetate (Fluka) for 1 min at room temperature. The samples were analyzed in a transmission electron microscope (CM12, Philips, Eindhoven, The Netherlands) equipped with a CCD camera (Ultrascan 1000, Gatan, Pleasanton, CA, USA) at an acceleration voltage of 100 kV.

### Immunofluorescence

Cells on coverslips were fixed in cold methanol for 3 min at −20°C or 2% paraformaldehyde in PBS for 10 min at room temperature. Coverslips were permeabilized for 5 min in PBS containing 0.1% Triton X-100. After blocking in 1% bovine serum albumin (BSA)-PBS for 30 min, the samples were incubated with primary and secondary antibodies (diluted in 1% BSA-PBS) and incubated for 45 min at room temperature in a humid chamber. When indicated nuclei were stained with DAPI. Images were acquired using a CLSM (Leica, DM 5500 Q) equipped with a 63×1.3 oil objective. Data were analyzed with Leica Application Suite (Mannheim, Germany), the Imaris software package (Bitplane, Switzerland) and Image J version 2.0.0-rc-48/1.50i (http://imagej.net/Fiji). Images were prepared for publication using PowerPoint (Microsoft) software.

For acquisition of tissue culture fluorescent images, a Carl Zeiss Axiovert S100 inverted fluorescence phase contrast trinocular microscope was used equipped with a 20X/0.30 Ph1 lens. Images were analyzed using AxioVision Rel 4.8 (06–2009)(Zeiss, Switzerland).

### Determination of numbers, areas, and frequencies of viroplasms

The viroplasms area and numbers were obtained using the “analyze particles tool” from Image J 1.48v (W. Rasband, NIH, USA. http://imagej.nih.gov/ij). Values, frequencies, and statistical analysis were performed with Microsoft® Excel® for MAC 2011Version 14.7.0.

### Determination of infectivity of viral progeny

At the indicated time post-infection, cells were trypsinized, harvested and centrifuged at 530 x g for 2 min. Cellular pellets were resuspended in 1 ml media, quantified and normalized to an equal number of cells for all the experimental conditions. The cells were centrifuged at 530 x g for 2 min and consecutively frozen in liquid nitrogen and thaw at 37°C in a water bath for three times. Then, the pellets were resuspended in 100μl of DMEM, centrifuged at 1200 x g for 5 min, and the supernatants were recovered. The virus supernatant was activated for 30 min with 5 μg/ml trypsin (from porcine pancreas, Sigma-Aldrich) at 37°C, and then two-fold serial dilutions were prepared. Viral titer was determined in NSP5-EGFP/MA104 cells, and unsynchronized cells were established as 100% of viral progeny, a method broadly described elsewhere [53–55].

### Eg5 perinuclear assay

After immunofluorescence for Eg5 detection, images were acquired using CLSM (Leica, DM 5500 Q) equipped with a 63X oil objective. Data were analyzed with Leica Application Suite (Mannheim, Germany) and the Imaris software package (Bitplane, Switzerland). The area of distribution of Eg5 (E) and nuclei (N) were determined using ImageJ 1.42 q (W. Rasband, NIH, USA. http://imagej.nih.gov/ij). The condensation of Eg5 to the perinuclear space was expressed as [(E-N)/N] ratio. Values and statistical analysis were performed with Microsoft® Excel ® for Mac 2011, version 14.5.1, using an unpaired two-tailed Student’s t-test.

### Immunoblotting

In general, cells were seeded in 12-well plates and lysed at the indicated time points in 30 μl lysis buffer (100 mM Tris-HCl, pH 8.0, 250 mM NaCl, 0.5% Nonidet P-40, cOmplete protease inhibitor (Roche) and Halt™ Phosphatase Inhibitor Single-Use Cocktail (Thermo Scientific)). The cell lysates were processed as described by Eichwald *et al*., 2004 [24].

## Results

### RV arrests the cell cycle in S/G2-phase independently of the viral strain

**Fig 1:**
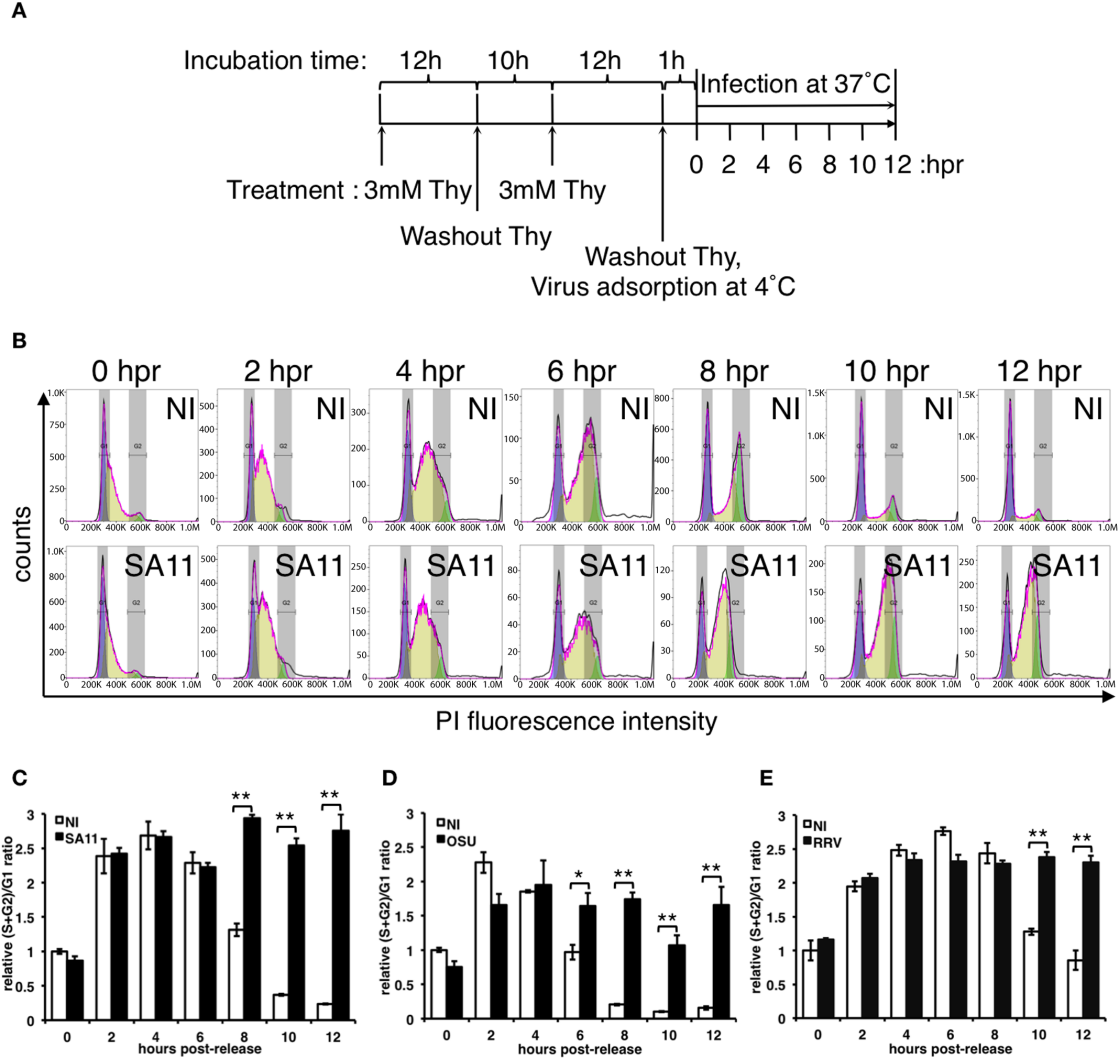
RV-infected MA104 cells get arrested in S/G2 phase in a strain-independent manner. (A) Schematic representation of the procedure used for cell synchronization by double blocking with thymidine (Thy) and further RV-infection. (B) Flow cytometer histograms of synchronized MA104 cells NI (upper row) or infected with simian RV strain SA11 [MOI, 25 VFU/cell] (lower row) at 0, 2, 4, 6, 8, 10 and 12 hours post-release (hpr) from thymidine block. The histograms overlay the DNA content obtained by the Watson model being purple, yellow and green areas under the curve of G1, S, and G2 phase percentage values, respectively. The plots show the relative (S+G2)/G1 ratio comparing NI and RV-infected [MOI, 25 VFU/cell] cells at each indicated time post-release from thymidine. As indicated, cells were infected with simian SA11 strain (C), porcine OSU strain (D) and simian RRV strain (E). The relative (S+G2)/G1 ratio was calculated considering NI at 0 hpr as a value of 1. Data represent the mean ± SEM from three independent experiments (t-test, *p<0.05; ** p<0.01).

We previously described that RV requires the MT-network to allow the formation, maintenance and perinuclear condensation of viroplasms. Additionally, MTs become acetylated in the neighborhood of the viroplasms, a process considered as a requirement for MT-stabilization [18]. Thus, RV replication requires an intact MT-network. During cell cycle progression, MTs get depolymerized at the onset of mitosis to form MT-spindles necessary for cell division. We hypothesized that RV infection could arrest cell cycle progression by impeding MT breakdown, thereby favoring its replication. To test this hypothesis, we synchronized MA104 cells in G1/S-phase through double-block with 3mM thymidine followed by RV-infection (simian SA11 strain). The cell cycle progression was monitored at 0, 2, 4, 6, 8, 10 and 12 hours post-release (hpr) by staining the cellular DNA content with propidium iodide (PI) (Fig 1A). As depicted in Fig 1B, immediately after release from the thymidine block (0 hpr) both non-infected (NI) and SA11-infected cells were in G1/S phase; this was also reflected in the finding of identical (S+G2)/G1 ratios (Fig 1C). As expected, at later times post-release (from 2 to 12 hpr) NI cells progressed into the cell cycle, reflected by a decrease of the relative (S+G2)/G1 ratio. However, SA11-infected cells were not able to progress, getting arrested in S/G2-phase (4 to 12 hpr), as visualized in both the cell cycle histogram (Fig 1B) and by the increase of the (S+G2)/G1 ratio when compared to NI cells (Fig 1C). It is worth mentioning that the increase in fluorescence intensity at the S/G2 phase of infected cells is due to DNA duplication and not to an increase of newly replicated viral dsRNA, as observed by fluorescence microscopy of PI stained infected cells (S1A Fig). A similar phenotype on cell cycle arrest in S/G2 was also observed in cells infected with porcine OSU (Fig 1D and S1B and S1D Fig) or simian RRV strains (Fig 1E and S1C and S1D Fig). Thus, the data strongly suggest that the RV infection halts the cell cycle progression before mitosis and that this happens independently of the RV strains used.

### RV infection induces cell cycle arrest independently of the cell type

**Fig 2:**
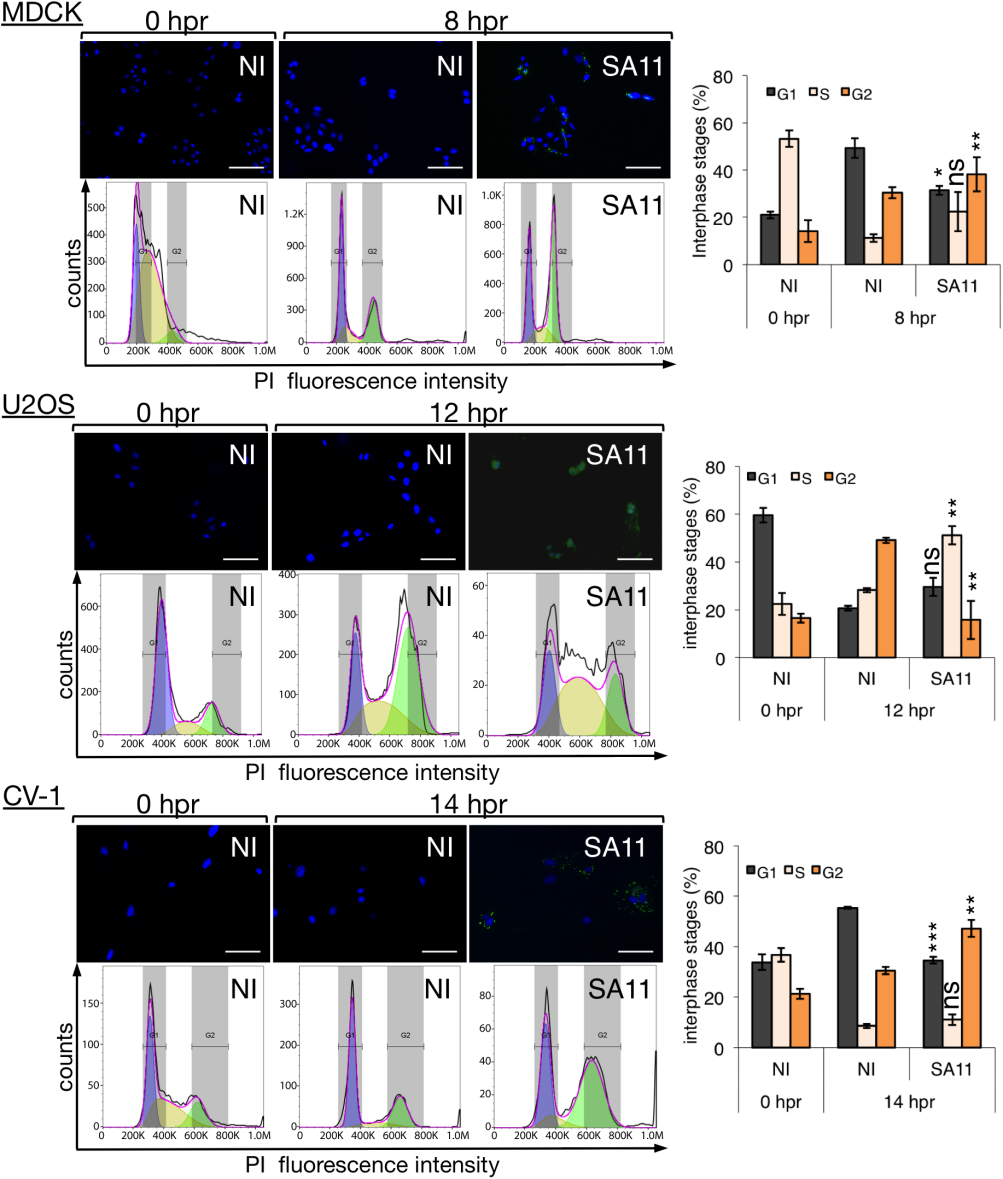
RV-infected cells get arrested in S/G2 phase in a cell type-independent manner. Flow cytometer histograms from double-thymidine synchronized MDCK (Madin-Darby canine kidney cells), U2OS (human bone osteosarcoma epithelial cells) and CV-1 (monkey kidney cells) cells infected with simian RV strain SA11 [MOI, 25 VFU/cell] and analyzed at 8, 12 and 14 hpr from thymidine. The selected time for analysis was dependent on a previous characterization of a whole replication cycle of each cell type. The histograms overlay the DNA content obtained by DJF mathematical model where purple, yellow and green areas under the curve correspond to the values of G1, S and G2 phases, respectively. An immunofluorescence picture, above each of the DNA content histograms, represents an immunostaining for viroplasms detection (mouse mAb anti-NSP5 [56], green) and stained for nuclei (DAPI, blue). Scale bar is 100μm. The plot, at the right of each panel, compares the percentage of the interphase stages (G1, S, and G2) from NI and SA11-infected cells at the indicated time post-release from thymidine. Each data correspond to the mean± SEM from three independent experiments. The SA11-infected cells compared with NI cells at the same time post-release; where ns, not significant p>0.05, (**) p<0.01 and (***) p<0.001 using two-tailed Student’s t-test.

Following this line of thought, we investigated whether RV infection also arrests the cell cycle in other cell lines. Several cell lines, such as canine kidney MDCK cells, human osteosarcoma U2OS cells or green monkey fibroblast CV-1 cells were synchronized in G1/S-phase by double blocking with thymidine. Depending on the nature of the cell lines synchronized with thymidine, at 0 hpr the cell cycle histogram can be visualized with most of the cells in G1/S phase (MDCK) or G1 (U2OS and CV-1). Cells were then RV-infected, and the cellular DNA content analyzed at the indicated hours post-release (Fig 2). The selected times post-release were based on previous cell cycle progression analysis for each independent cell line. Since after synchronization, not all the cell lines tested showed a sharp peak in G1 phase as MA104 cells do, the relative (S+G2)/G1 ratio could not be used. Hence we present the percentage values of each interphase stage. The results show that all the cell lines analyzed were arrested in S/G2 phase following RV infection as denoted by both the DNA content histograms and the percentage of the interphase stages plot compared to NI cells. These data strongly indicate that RV can arrest cells at the onset of mitosis independently of the cell type background.

### RV induction of cell cycle arrest in S/G2 phase enhances viral replication

**Fig 3:**
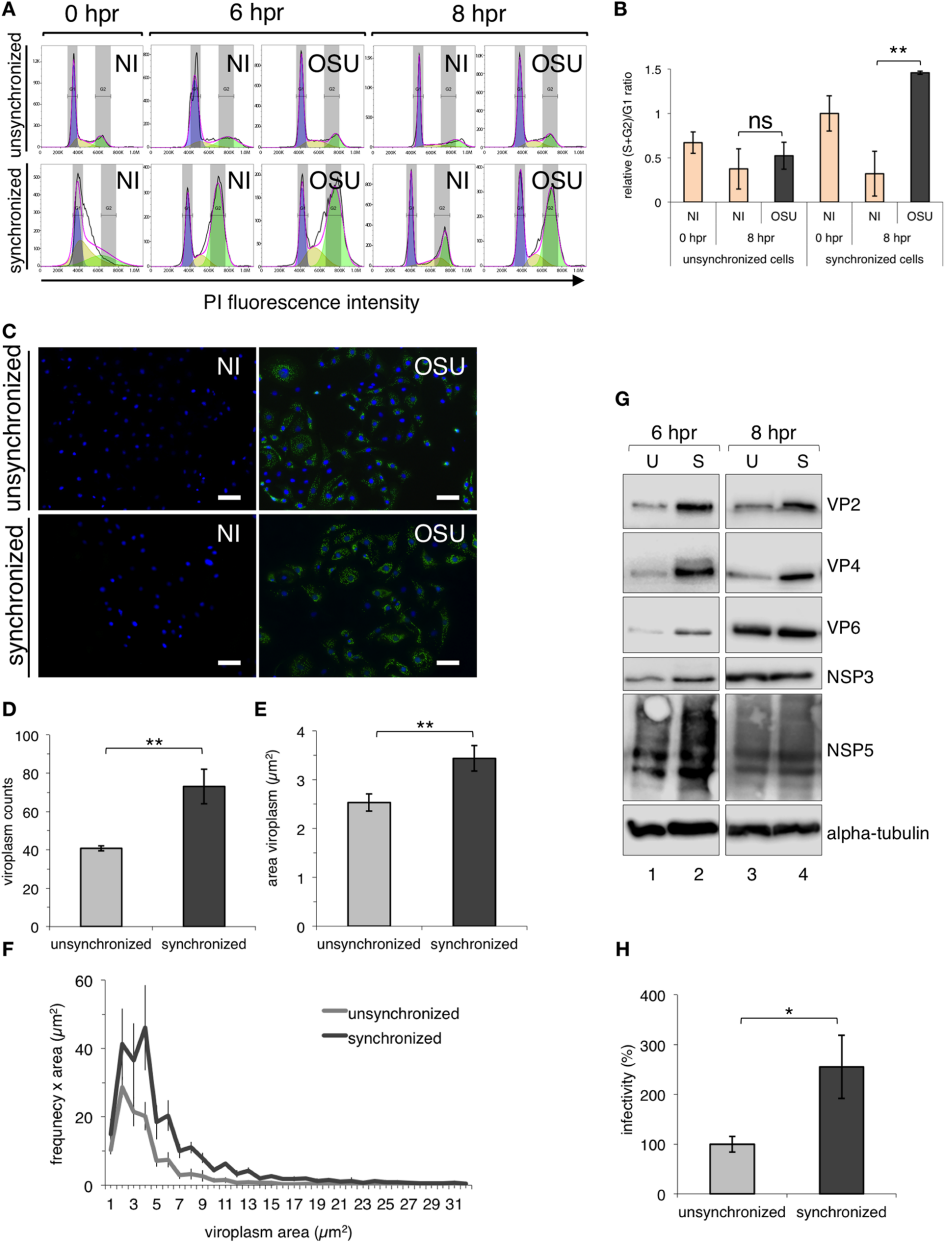
The cell cycle arrest induced by RV in S/G2 phase improves viral replication. Unsynchronized and synchronized MA104 cells were infected with porcine RV strain OSU [25 VFU/cell] and analyzed at 0, 6 and 8 hpr from double thymidine block. (A) Representative flow cytometer histograms of unsynchronized (upper panel) and synchronized (lower panel) MA104 cells NI or infected with porcine RV OSU strain [MOI, 25 VFU/cell]. The histograms overlay the DNA content obtained by the DJF model where purple, yellow and green areas under the curve correspond to the values of G1, S and G2 phases, respectively. (B) The plot for relative (S+G2)/G1 ratio from comparing NI and OSU-infected unsynchronized and synchronized cells at 0 and 8 hpr from thymidine. The relative (S+G2)/G1 ratio was calculated considering synchronized NI at 0 hpr as a value of 1. Data represent the mean ± SEM from four independent experiments (**) p<0.01 and ns p>0.05). (C) Immunofluorescence of unsynchronized and synchronized cells NI and SA11 infected at 8 hpr from thymidine. The merged images are immunostaining for viroplasms detection (mouse mAb anti-NSP5, green) and nuclei staining (DAPI, blue). Scale bar is 100 μm. The number (D) and size (E) of viroplasms per cell plotted at 8 hpr from thymidine for unsynchronized and synchronized cells. The data represented the mean ± SEM of four independent experiments, t-test, (**) p<0.01; number >150 cells. (F) Distribution of the viroplasm frequency in unsynchronized and synchronized cells at 8 hpr. (G) Immunoblotting from a normalized number of unsynchronized (U, lanes 1 and 3) and synchronized (S, lanes 2 and 4) cells at 6 (lanes 1 and 2) and 8 hpr (lanes 3 and 4). VP2, VP4, VP6, NSP3 and NSP5 viral proteins were detected using anti-VP2, anti-VP5, anti-RV, anti-NSP3 and anti-NSP5 antibodies, respectively. Alpha-tubulin was used as loading control. (H) At 8 hpr, RV-infected unsynchronized and synchronized cells were count normalized, lysed and viral titer was determined. Data are expressed as the percentages of infectivity obtained from unsynchronized cells, which is considered as 100% of infectivity. The data is the mean ± SEM of three independent experiments, t-test, (*) p<0.05.

We next asked whether induction of an arrest in S/G2 phase of the host cell could provide an advantage to the RV life cycle. To evaluate this, we compared RV infection in unsynchronized and synchronized MA104 cells. For this purpose, we examined NI and OSU-infected MA104 cells synchronized in G1/S phase or unsynchronized. The DNA content of NI and RV-infected cells was compared in unsynchronized and synchronized cells at 6 and 8 hpr (Fig 3A). The cell cycle arrest induced by RV in S/G2 phase was clearly distinguishable when comparing synchronized with unsynchronized cells, as indicated by the DNA content plots and the relative (S+G2)/G1 ratios (Fig 3B). Under these same conditions, viral infection was also monitored at 8 hpr by immunofluorescence for the detection of viroplasms with an anti-NSP5 antibody (Fig 3C). We observed an apparent increase in the number of viroplasms per cell in synchronized cells, compared to unsynchronized ones. To confirm this observation, we determined the number and area of viroplasms per cell at 8 hpr in both conditions (Fig 3D and 3E). Interestingly, the number of viroplasms (Fig 3D) was at least duplicated when cells were synchronized. Also, the area of individual viroplasms was larger in synchronized cells (Fig 3E). A comparable result was obtained when analyzing the size of viroplasms, plotting the distribution of the size frequency in infected unsynchronized or synchronized cells (Fig 3F). We found that viroplasms from synchronized cells were more numerous and bigger than in unsynchronized cells. Upon these conditions, we examined the level of viral proteins in normalized unsynchronized or synchronized cells at 6 and 8 hpr. For this purpose, cell lysates from equal amounts of cells were prepared and assessed by immunoblotting. Remarkably, at 6 hpr (Fig 3G, lanes 1 and 2) all viral proteins analyzed (VP2, VP4, VP6, NSP3, and NSP5) enhanced their expression in synchronized cells. This effect was also observed at 8 hpr for VP2 and VP4, while no difference in expression was observed for VP6, NSP3, and NSP5 (Fig 3G, lanes 3 and 4). The amounts of alpha-tubulin were similar in all lysates. Also, we analyzed virus yields from normalized RV-infected unsynchronized or synchronized cells (Fig 3H). We found an increase in infectivity of the viral progeny when cells were synchronized compared to unsynchronized cells. Collectively, these data strongly suggest that the induction of the cell cycle arrest by RV in S/G2 phase favors RV replication by increasing the number and size of viroplasms and enhancing viral protein translation and formation of viral progeny.

### RV induces depletion of cyclin B1

**Fig 4:**
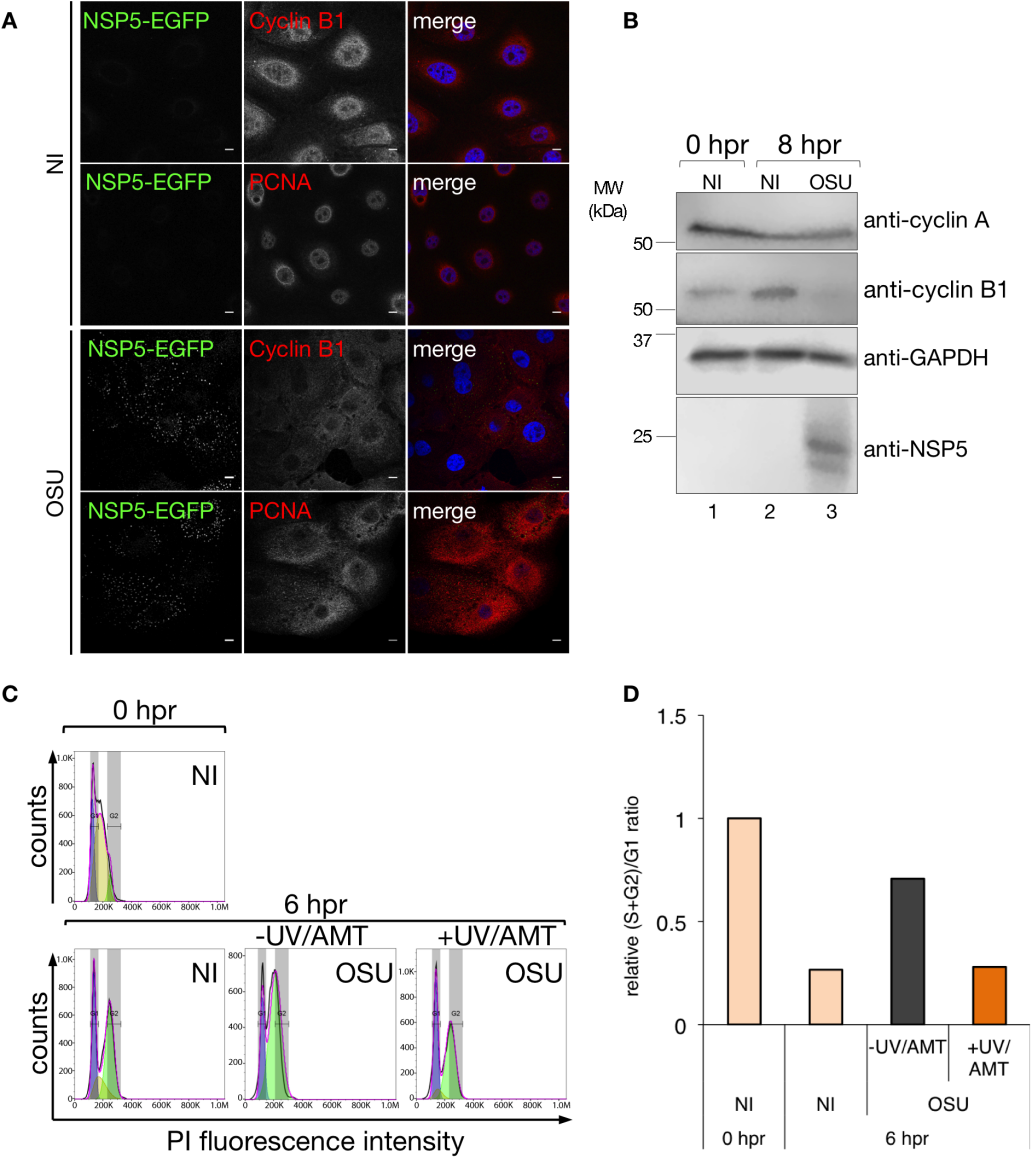
RV depletes cyclin B1 and replication as a condition for the cell cycle arrest. (A) Immunofluorescence of NI or OSU-infected [MOI, 25 VFU/cell] synchronized NSP5-EGFP/MA104 cells at 6 hpr. Cells were fixed in paraformaldehyde and immunostained for cyclin B1 (mouse mAb anti-cyclin B1, red) or PCNA (mouse mAb anti-PCNA, red), viroplasms detected with NSP5-EGFP (green) and nuclei stained with DAPI (blue). A merged image is presented in the right column. Scale bar is 10μm. (B) Immunoblotting of cell lysates from synchronized MA104 cells NI or OSU-infected [MOI, 25 VFU/cell] at 0 and 8 hpr from thymidine for the detection of cyclin A, cyclin B1, and NSP5. GAPDH is the loading control. The molecular weight markers are indicated. (C) Porcine OSU strain TLPs [MOI, 25 VFU/cell] inactivated with UV/psoralen (UV/AMT). Thymidine-synchronized MA104 cells were NI or infected with transcriptionally active (-UV/AMT) or inactive (+UV/AMT) virus. Cell cycle histograms for 0 hpr (top panel) and 6 hpr (bottom panel) are presented. The histograms overlay the DNA content obtained by DJF model, where purple, yellow and green areas under the curve correspond to the values of G1, S, and G2 phases, respectively. (D) The plot representing the relative (S+G2)/G1 ratio comparing NI cells with active (-UV/AMT) or inactive (+UV/AMT) TLPs at 6 hpr.

Cell cycle progression is regulated and monitored by several checkpoints, verifying the phase process and repairing damaged DNA [27]. The checkpoints are mainly controlled by cyclins and CDKs [28]. At the onset of mitosis, following the nuclear envelope breakdown, A-type cyclins are degraded facilitating the formation of CDK1-cyclin B complexes responsible for driving cells through mitosis [27]. To elucidate the localization and expression of some of these critical cell cycle regulators upon RV infection, we investigated the localization of cyclin B1 by immunofluorescence. As depicted in Fig 4A, cyclin B1 localized in the perinuclear and nuclear region at 6 hpr in NI synchronized MA104 cells stably expressing NSP5-EGFP fusion protein (NSP5-EGFP/MA104) cells. A similar result was obtained with the control protein PCNA (proliferation cell nuclear antigen). PCNA is a master regulator of the coordination processes of maintenance, duplication, and transmission of the genome during mitosis [57] and therefore is present in the nuclear region at the onset of mitosis. However, upon RV infection with both porcine OSU strain (Fig 4A) or simian RRV strain (S2A Fig) cyclin B1 was poorly expressed and was found diffused in the cytosol. As expected, PCNA was unable to localize in the nucleus, being found distributed throughout the cytosolic region. In fact, cyclin B1 was depleted upon RV infection as shown by immunoblotting in cell lysates from synchronized MA104 cells at 8 hpr and compared to NI cells (Fig 4B). Similarly, also cyclin B1 was depleted in OSU-infected synchronized Caco-2 cells, thus showing that RV infection also arrests Caco-2 cells in S/G2 phase (S2B, S2C and S2D Fig). Our data indicate that RV-infected cells do not enter mitosis, suggesting that RV subverts the cell-cycle checkpoint.

### RV cell cycle arrest is dependent on active viral replication

With the aim to identify the virus life cycle step involved in cell cycle arrest, we inactivated RV virions with UV/psoralen, rendering the viral particles transcriptionally incompetent but fit for cell penetration [52, 58]. After inactivation, the virion integrity was verified by transmission electron microscopy using negative staining (S2E Fig) while the inability to form viroplasms, and thus to replicate, was checked after infection of NSP5-EGFP/MA104 cells. The cellular DNA content of MA104 cells synchronized in G1/S phase was assessed at 6 hpr after infection with replication competent or UV/psoralen-inactivated RV virions. As depicted in Fig 4C, cells infected with active-RV (-UV/AMT) were arrested in S/G2 phase, while those NI or infected with inactive-RV (+UV/AMT) were able to proliferate. The decrease of the (S+G2)/G1 ratio in NI cells or cells infected with inactive-RV at 6 hpr was consistent with cell cycle progression when compared to NI cells at 0 hpr. As expected, the relative (S+G2)/G1 ratio in cells infected with active-RV was higher than in cells infected with inactive ones (Fig 4D). Altogether, these data show that RV-cell penetration is a step upstream of the cell cycle arrest and that viral replication is essential to accomplish the cell cycle arrest.

### Identification of pathways involved in the RV cell cycle arrest

**Fig 5:**
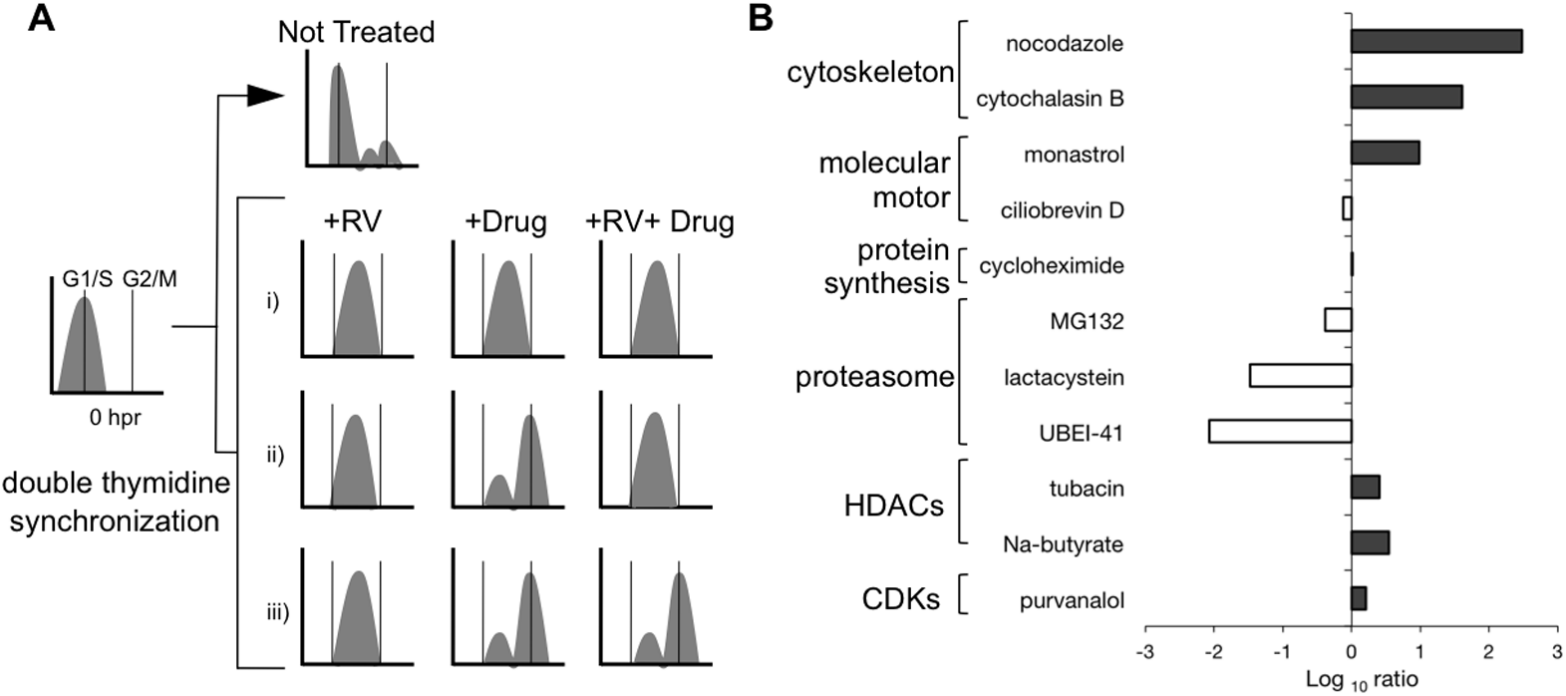
Actin, MTs, and kinesin are involved in the RV arrest of the cell cycle. (A) An experimental model targeting host pathways by specific drug inhibitors directly related to RV-arrest of the MA104 cell cycle. At 8 hpr, flow cytometer histograms from RV-infected synchronized cells compared with those from both NI and RV-infected cells after adding the drug at 30 minutes post-release from thymidine to determine a reverting drug for the RV-induced arrest at S/G2 phase. Thus, three conditions can be expected: (i) the drug intrinsically arrests in S-phase, (ii) the drug cannot interfere with RV-arrest and (iii) the drug reverts the RV-arrest in S-phase. (B) Plot showing the Log10 ratio of the drugs for either not affecting (white bars) or reverting (gray bars) RV cell cycle arrest. The drugs grouped into the major inhibitory physiological pathways such as cytoskeleton, the molecular motor, protein synthesis, proteasome, deacetylases and cyclin-dependent kinases. Each set of data corresponds to the average of two independent experiments. The concentration for each of the drugs used in this assay was: 10μM nocodazole, 10 μM cytochalasin B, 10μM monastrol, 50μM ciliobrevin D, 100μg/ml cycloheximide, 10μM MG132, 10μM lactacystin, 10μM UBEI-41, 10μM tubacin, 5mM Na-butyrate and 10μM purvalanol A.

To identify host factors involved in RV cell cycle we next chose a set of drugs (Fig 5B) that specifically target cytoskeleton, molecular motors, protein-synthesis, proteasomal activity, histone deacetylases (HDACs) and CDKs. We set up an experimental design that compares DNA content of synchronized RV-infected cells treated with drugs with that of non-treated cells. For each of the tested drugs, a ratio value was obtained that when positive indicated a reversion of the RV-cell cycle arrest by the drug. Depending on the effect of the drug in non-infected versus infected cells different outcomes are possible and schematically shown in Fig 5A. Only those drugs that prevented RV from arresting in S-phase (case (iii) in Fig 5A) were further considered. Noteworthy and as denoted in S3 Fig, all the drugs used in this assay, at the indicated concentration and time of treatment, do not have any cytotoxic effect.

The drugs that had an effect on reverting the RV-arrest were the cytoskeleton inhibitors nocodazole and cytochalasin B; the allosteric inhibitor for kinesin Eg5 monastrol; the HDACs inhibitors tubacin and Na-butyrate and the CDKs inhibitor purvalanol A. All these drugs allowed progression of the cell cycle upon RV infection (Fig 5B) suggesting that MT and actin networks, kinesin Eg5, HDACs, and CDKs are directly involved in upstream pathways implicated in the RV-induced arrest.

### Kinesin Eg5 delocalizes in viroplasms of RV-infected cells

**Fig 6:**
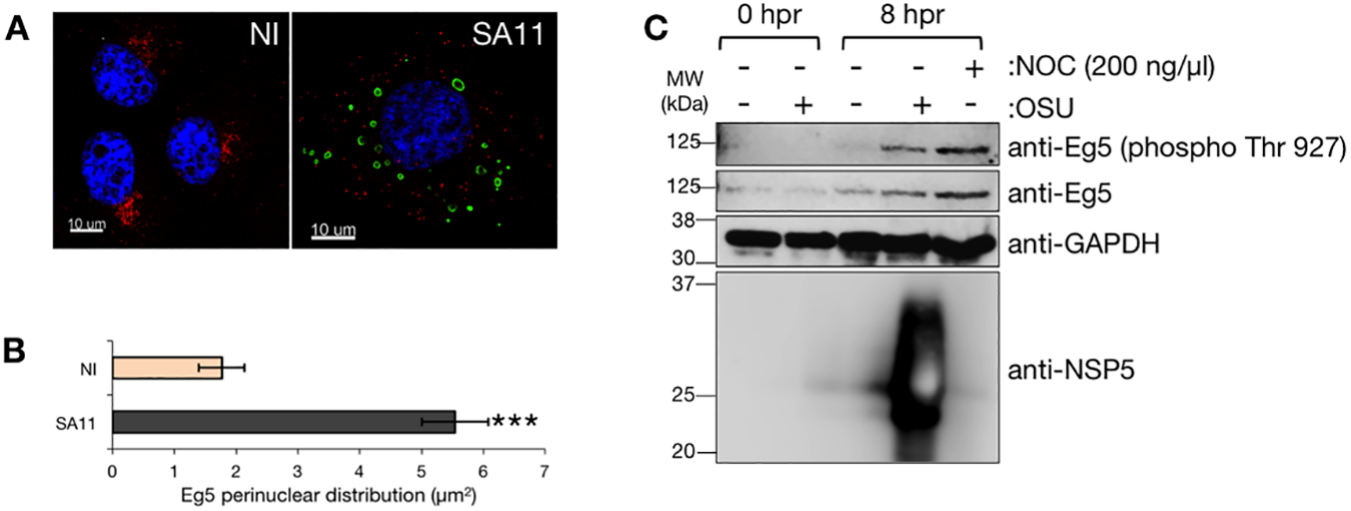
RV subverts kinesin motor Eg5. (A) Immunofluorescence of NI or SA11-infected [MOI, 25 VFU/cell] synchronized MA104 cells at 8 hpr, for the visualization of Eg5 (anti-Eg5, red), viroplasms (anti-NSP5, green) and nucleus (DAPI, blue). Scale bar is 10 μm. (B) Eg5 perinuclear area determined for NI and SA11-infected synchronized MA104 cells at 8 hpr. Data represents the mean ± SEM (t-test, *** p<0.001, n≥30). (C) Immunoblotting of cell lysates at 0 and 8 hpr from NI and OSU-infected [MOI, 25 VFU/cell] synchronized MA104 cells. Viral infection detected with an anti-NSP5 specific antibody; phosphorylated Eg5 and Eg5 detected with rabbit anti-Eg5 (phosphoThr927) and mouse mAb anti-Eg5, respectively. GAPDH staining was used as loading control. Protein molecular weights are indicated. The phosphorylated-Eg5 positive control corresponds to cells treated post-synchronization with nocodazole (200ng/μl) [60].

We have previously shown that viroplasms require kinesin Eg5 for its formation, maintenance and perinuclear condensation [18]. As described above, monastrol, an allosteric inhibitor for Eg5, can revert RV arrest of the cell cycle (Fig 5B). Thus, our outcomes indicate a link between viroplasm formation and the arrest of the cell cycle. During the cell interphase, Eg5 polarizes in a condensed zone near the pericentriolar material. However, at the onset of mitosis Eg5 gets phosphorylated and localizes in the mitotic spindles where it is required for the maintenance of the spindle bipolarity, which occurs at the time of entry into mitosis [31, 59]. To clarify the role of Eg5 in RV replication, we investigated the localization of Eg5 by immunofluorescence (Fig 6A) in RV-infected synchronized cells. Surprisingly, at 8 hpr Eg5 was vastly distributed in the cytosol in RV-infected cells, which was in marked contrast to its pericentriolar localization in NI cells as denoted by the quantification of the Eg5 perinuclear distribution (Fig 6A and 6B). Since Eg5 gets phosphorylated in Thr 927 once it localizes at the mitotic spindles [31], we then investigated the phosphorylation status of Eg5 upon RV-infection. Cell lysates from synchronized RV-infected MA104 cells were prepared at 8 hpr and compared with extracts from NI cells at 0 and 8 hpr (Fig 6C). In agreement with Smith *et al*., 2011 [59], we detected lower expression of Eg5 at 0 hpr corresponding to cells synchronized in G1 phase. The MT-depolymerizing agent nocodazole was used to synchronize cells in G2/M phase to permit the detection of Eg5 phosphorylation in Thr927 [59, 60]. The data show that Eg5 was phosphorylated in RV-infected lysates to a similar extent than in synchronized cells treated with nocodazole, but not in lysates from NI cells at both 0 and 8 hpr or RV-infected cells at 0 hpr. Thus, our results indicate that in the presence of replicating RV, Eg5 was both re-distributed from its pericentriolar localization into viroplasms and phosphorylated at Thr927.

### NSP3, NSP5, and VP2 play a role in RV-induced cell cycle arrest

**Fig 7:**
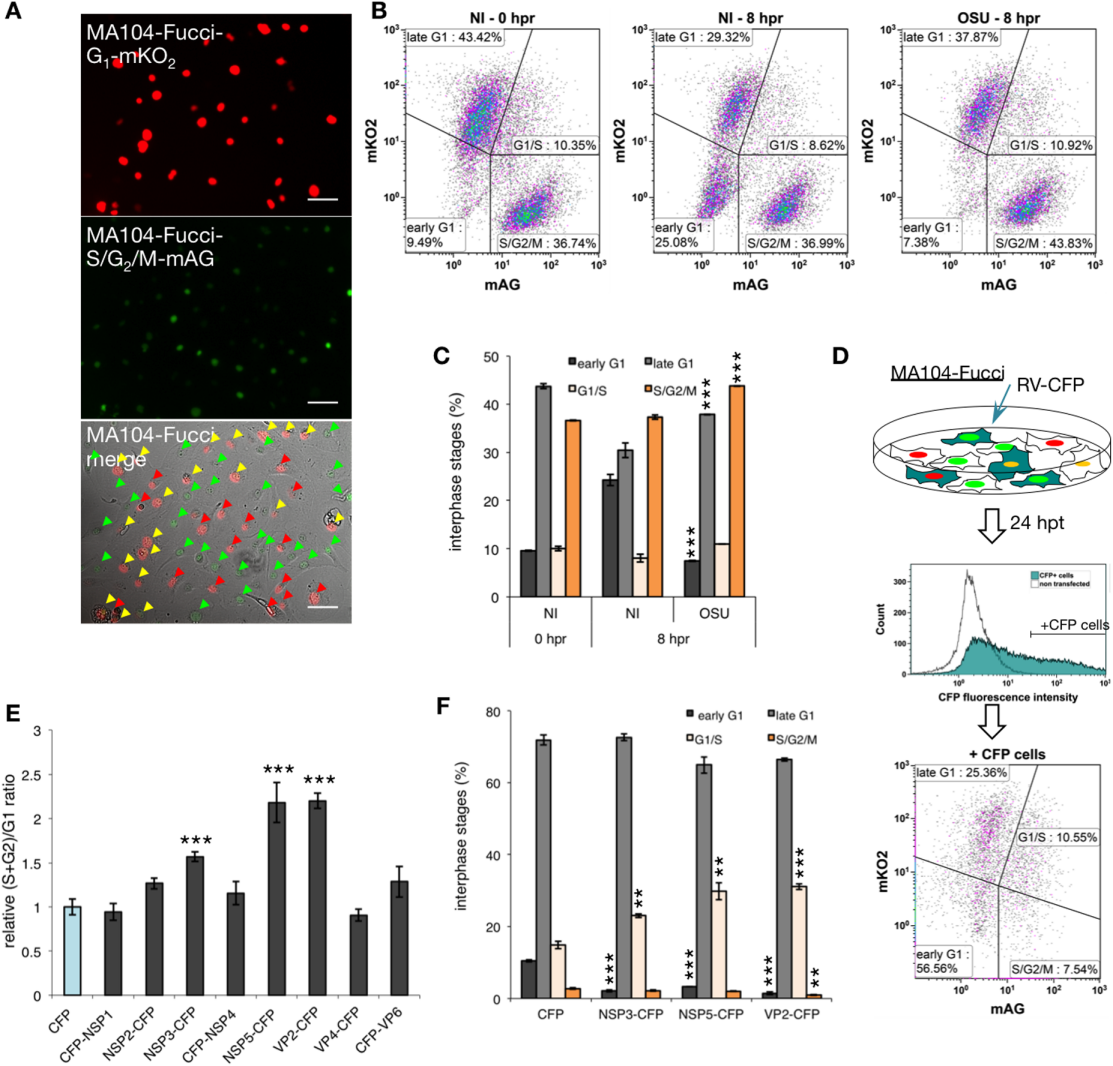
NSP3, NSP5, and VP2 play a role in the RV cell cycle arrest. (A) Fluorescence microscopy of non-synchronized MA104-Fucci cells showing human Cdt1 (aa 30–120) fused to orange fluorescent monomeric Kusabira-Orange 2 (G1-mKO2) (red, top image), human Geminin (aa 1–110) fused to green fluorescent monomeric Azami-Green1 (S/G2/M-mAG, green)(middle image) and the fluorescence merge with bright field (bottom image). Red, yellow and green arrowheads indicate cells in early/late G1, G1/S, and S/G2/M phases respectively. Scale bar is 100μm. (B) Density plot of NI or OSU-infected [MOI, 25 VFU/cell] synchronized MA104-Fucci cell populations at 0 and 8 hpr. The population percentages, discriminated by its mAG and mKO2 fluorescence intensities, were gated for late G1, early G1, G1/S and S/G2/M. (C) Interphase stages plot of NI and OSU-infected synchronized MA104-Fucci cells at 0 and 8 hpr. Data are represented as the mean ± SEM (t-test, ***p<0.001, n = 3). (D) Experimental model for determination of RV protein(s) responsible for the cell cycle arrest. MA104-Fucci cells were transfected with RV proteins fused to CFP at their N- or C-terminus. Cells were harvested at 24 hpt and immediately analyzed by flow cytometry. The +CFP cells were gated, and interphase stages were analyzed using the Fucci system. (E) Plot showing relative (S+G2)/G1 ratio of RV proteins fused to CFP in MA104-Fucci at 24 hpt. Each RV protein fused to CFP was tested for functional expression by fluorescence microscopy at 24 hpt (data not shown). The relative (S+G2)/G1 ratio was calculated considering CFP only cells as value of 1. Data are represented as the mean ± SEM (t-test, *** p<0.001, n = 3). (F) Plot showing the percentage of the interphase stages of MA104-Fucci transfected with the indicated proteins fused to CFP. Data are represented as the mean ± SEM (t-test, ** p<0.01, *** p<0.001, n = 3).

To identify RV protein(s) involved in cell cycle regulation, we developed a Fucci (fluorescent ubiquitination-based cell cycle indicator)-system in MA104 cells based on the overexpression of the fusion proteins Cdt1-mKO2 (red) and geminin-mAG (green) that during the cell cycle become ubiquitinated and targeted to proteasomal degradation. As a result, cells appear red throughout the G1 phase and green throughout S, G2, and M phases. During the G1/S transition, when Cdt1 levels decrease and those of geminin increase, both proteins are present, and cells fluoresce yellow. Synchronized MA104-Fucci cells presented a replication cycle of 8 hpr and the progression followed by flow cytometry correlated to its DNA content as denoted by the same (S+G2)/G1 ratio (Fig 7A and S4A to S4E Fig). As expected, we observed that upon infection with OSU at 8 hpr these cells were arrested in S/G2 (Fig 7B and 7C and S5A Fig). These results were confirmed by analysis of DNA content (S5B and S5C Fig). We then transfected MA104-Fucci cells with plasmids expressing RV proteins (NSP1, NSP2, NSP3, NSP4, NSP5, VP2, VP4, and VP6) fused to CFP (cyan fluorescent protein) to determine those responsible for mediating cell cycle arrest. Cells were analyzed at 24 hpt by flow cytometry gating on positive CFP cells and compared to cells transfected with CFP only (Fig 7D). The cell cycle was easily identified based on the Fucci system. The data (Fig 7E and S5C Fig) analyzed as (S+G2)/G1 ratio, indicated that only cells expressing NSP3-CFP, NSP5-CFP and VP2-CFP were able to arrest cell cycle progression in MA104-Fucci cells significantly. Interestingly, the arrest induced by these three proteins was mainly centered in G1/S (Fig 7F).

Since most of the high-tuned regulation of the cell cycle progression is dependent on phosphorylation cascades [61] and one of the identified proteins (NSP5) shows a hyperphosphorylated pattern [62, 63], mediated by essential kinases involved in the cell progression (such are CK1α (casein kinase 1 alpha) [24], casein kinase 2 [64] or protein kinase C [65]). We hypothesized that the hyperphosphorylated form of NSP5 could deregulate the activity of the kinases mentioned above. We have previously shown that NSP5 hyperphosphorylation is triggered by CK1α, via the phosphorylation of a non-canonical motif that involves NSP5 serine 67 [24]. Here, we fused CFP to NSP5 mutants carrying point mutations in S67 (S67A and S67D) (S5E Fig) and expressed them in MA104-Fucci cells. As depicted in S5F and S5G Fig, all the tested NSP5 versions were able to significantly arrest the cell cycle in the infected cells mainly at G1/S phase, thus indicating that NSP5 hyperphosphorylation is not required for the cell cycle arrest.

## Discussion

A common strategy used by DNA or RNA viruses is the subversion of the host cell cycle by arrest, which generates resources and conditions that favor replication of the infectious virus. Several reasons have been proposed to explain the link between the cell cycle arrest with RNA viruses, including i) increase in the efficiency of the viral replication and translation; ii) improvement in virus assembly and iii) a delay of apoptosis of the infected cells [66]. In this study, we present strong evidence indicating that RV infection arrests the host cell cycle in S/G2 phase. Our data were confirmed using synchronized cells stained for their DNA content and through the Fucci system in MA104 cells. Importantly, we found that RV infection can arrest cells in S/G2 phase independently of the viral strain and the nature of the host cell (Figs 1 and 2). Thus, RV intercepts a common pathway in the host cell that is shared by all tested RV strains. We show that the RV-induced arrest in S/G2 phase enhances virus replication resulting in a rise of viral progeny (Fig 3). RV replication may be favored as a consequence of the S/G2 arrest like i) stabilization of the MT-network with a positive effect on viroplasms; ii) prevention of early cell death, already reported in RV-infected cells [67–70]; iii) efficient virion assembly as the final stages occur in the endoplasmatic reticulum compartment [71], which gets disrupted during mitosis; iv) use of cap-binding proteins and cap-dependent translation (translation of all RV mRNAs is cap-dependent) that are impaired during mitosis and v) increased ribonucleotides levels that are required for viral genome synthesis [66, 72].

Our data also indicate that RV replication is more efficient in synchronized cells (Fig 3). Although the exact nature of the pathway targeted during RV infection is not presently known, we provide evidence indicating that it involves depletion of cyclin B1, which is required for the progression of the cell cycle into mitosis through the cyclin B1/CDK1 complex (Fig 4). We were able to revert the RV arrest by using drugs that interfere with both the actin and MT-networks and the motor kinesin Eg5, linking the requirement of a stabilized MT-network for viroplasm formation with the arrest of the cell cycle and hence, with RV replication (Fig 5). We previously showed that the viroplasms need of Eg5 for its formation and structure maintenance [18]. Here, using synchronized cells, we confirmed that Eg5 localizes throughout the cytosol and mediates its phosphorylation in Thr927 during the interphase, in a process likely mediated by CDK1 (Fig 6).

Cell cycle arrest was dependent on active viral replication rather than on virus entry (Fig 4) [52, 58]. Also, we identified three viral proteins (NSP3, NSP5, and VP2) capable of inducing arrest of cells in G1/S-phase (Fig 7). These results are in line with previous reports in which these viral proteins associate with the mastering of the viral life cycle [5, 9]. For example, NSP3 has an inhibitory effect on translation of cellular proteins due to its interaction with eIF4G and its participation in the re-localization of PABP and poly(A)-containing mRNAs to the nucleus [4, 5, 7, 73, 74]. Additionally, knocking down the phosphoprotein NSP5 in RV-infected cells impairs formation of viroplasms [75]. Likewise, the main core-shell protein VP2, besides its structural function protecting the viral genome, is also able to activate and regulate the RdRp VP1 protein, permitting genome replication [76]. VP2 is a main component of the viroplasms and is involved in the relocalization toward the perinuclear region [18, 21, 45]. The evidence presented here suggests that the cell cycle arrest mediated by RV is the consequence of the activity of more than one viral protein. The precise role of these RV proteins in the induction of the cell cycle arrest remains yet to be clarified and is subject to current research in our laboratory.

## Supporting information

S1 Fig Viroplasms not stained by PI and controls for cell cycle arrest with RV strains, OSU and RRV. (A) Immunofluorescence of non-infected (NI) and SA11-infected MA104 cells at 6 hpi. Cells were ethanol fixed for 3 min at -20°C and immunostained for viroplasms with a specific anti-NSP5 antibody (green) and nuclei stained with PI (red). Scale bar is 10μm. Representative flow cytometry histograms of synchronized MA104 cells NI and infected [MOI, 25 VFU/cell] with porcine OSU (B) or simian RRV (C) strains acquired at 0, 2, 4, 6, 8, 10 and 12 hpr from thymidine. (D) Immunofluorescence for viral infection of cell examined for DNA content. Each histogram overlays the DNA content by Watson mathematical model where purple, yellow and green areas under the curve correspond to the percentage values of G1, S and G2 phases, respectively. Cells were immunostained for viroplasms detection (anti-NSP5, green) and stained for nuclei (DAPI, blue). Scale bar is 100 μm.
(TIF)

S2 Fig Detection of cyclin B1 and PCNA in RRV-infected cells, RV-infected synchronized Caco-2 cells and EM of inactivated RV virions. (A) Immunofluorescence of RRV-infected [MOI, 25 VFU/cell] synchronized NSP5-EGFP/MA104 cells at 6 hpr. Cells were fixed in paraformaldehyde and immunostained for cyclin B1 (mouse mAb anti-cyclin B1, red) or PCNA (mouse mAb anti-PCNA, red), viroplasms detected with NSP5-EGFP (green) and nuclei stained with DAPI (blue). The merged image is presented in the right column. Scale bar is 10μm. (B) Flow cytometer histograms of synchronized Caco-2 cells (Human colon adenocarcinoma cells) infected with porcine OSU strain [MOI, 25 VFU/cell] and analyzed at 0, 2, 4, 6 and 8 hpr from thymidine. Each histogram overlays the DNA content by DJF mathematical model where purple, yellow and green areas under the curve correspond to the values of G1, S and G2 phases, respectively. (C) Plot showing the percentage of the interphase stages (G1, S, and G2) from synchronized non-infected (NI) and OSU-infected Caco-2 cells at the indicated times post-release from thymidine. (D) Immunoblotting of cell lysates from OSU-infected (lanes 1 to 5) and non-infected (lanes 6 to10) synchronized Caco-2 cells. The cells were harvested at 0, 2, 4, 6 and 8 hpr. Cyclin B1, cdc2-P (Tyr 15) and NSP5 were detected using specific antibodies. GAPDH used as loading control. The molecular weight markers are indicated. (E) Images of electron microscopy of negatively stained OSU-TLPs after inactivation with UV-psoralen (UV/AMT). Scale bar is 100 nm.
(TIF)

S3 Fig Characterization of RV-infected cells treated with drugs. MA104 cells were infected with OSU [MOI, 25 VFU/cell] and treated at 30 min post-infection with the indicated drugs. At 8 hpi, cells were fixed, immunostained for viroplasms detection (anti-NSP5, green) and stained for nuclei (DAPI, blue). Scale bar is 100 μm. The concentration of the drugs used was: 10μM nocodazole, 10 μM cytochalasin B, 10μM monastrol, 50μM ciliobrevin D, 100μg/ml cycloheximide, 10μM MG132, 10μM lactacystin, 10μM UBEI-41, 10μM tubacin, 5mM Na-butyrate and 10μM purvalanol A. The tested drugs, at the indicated incubation time and concentration, do not induced detectable cytotoxic effect.
(TIF)

S4 Fig Characterization of MA104-Fucci cells. Characterization of synchronized MA104-Fucci cells at 0, 4 and 8 hpr from thymidine. (A) Fluorescence microscopy. Each image corresponds to the fluorescence merge from Ctd1-mKO2 (red), Geminin-mAG (green) and a bright field. The red, yellow and green arrowheads indicate the cells in early/late G1, G1/S and S/G2/M phases, respectively. Scale bar is 100μm. (B) Density plots.The cells were discriminated by its Ctd1-mKO2 (red) and Geminin-mAG (green) fluorescence intensities and gated as early G1, late G1, G1/S and S/G2/M. (C) DNA content histograms determined by PI fluorescence intensity. The data were obtained using DJF model where purple, yellow and green areas under the curve correspond to the values of G1, S and G2 phases, respectively. G1 and G2 phases were constrained. (D) Interphase stages plot (early/late G1, G1/S and S/G2/M). (E) Comparison plot of relative (S+G2)/G1 ratio obtained from flow cytometry of fluorescence intensities of Ctd1-mKO2 and Geminin-mAG (gray bars) or DNA content of PI fluorescence intensity (orange bars). The relative (S+G2)/G1 ratio was calculated considering NI cells at 0 hpr as a value of 1. Data represented the mean ± SEM, from three independent experiments.
(TIF)

S5 Fig RV-infected synchronized MA104-Fucci and RV-CFP proteins expression. Characterization of NI and OSU-infected [MOI, 25 VFU/cell] synchronized MA104-Fucci cells after 0 and 8 hpr from thymidine. (A) Immunofluorescence of NI (upper row) and OSU-infected (lower row) synchronized MA104-Fucci cells. Cells were immunostained at 8 hpr for viroplasms detection (anti-NSP5, magenta, left column). Fucci sensors G1-mKO_2_ (red) and S/G2/M-mAG (green) are indicated (middle columns). A merged image is shown in the right column. Scale bar is 100 μm. (B) DNA content determined by PI fluorescence intensity. The cell cycle was calculated using DJF mathematical model where purple, yellow and green areas under the curve are the percentage values of G1, S and G2 phases, respectively. G1 and G2 phases were constrained. (C) The plot of relative (S+G2)/G1 ratio 0 and 8 hpr. (D) Immunoblotting of cell lysates of MA104 expressing RV proteins fused to CFP. The cells were transfected in the presence of VVT7.3 and lysed at 16 hpt. The membrane was incubated with anti-GFP antibody followed by the corresponding secondary antibody conjugated to peroxidase. The red dot and white arrows indicate the expected migration position for monomeric and oligomeric forms of each of the fusion proteins. The protein molecular markers are shown. The CFP tag was added at the N- or C-terminus of each RV protein as described elsewhere. Each RV protein fused to CFP was tested for functional expression by fluorescence microscopy at 24 hpt (data not shown). (E) Immunoblotting of cell lysates of MA104 expressing CFP (lane 1), NSP5-CFP wt or NSP5-CFP carrying the point mutations S67A and S67D (S63, 65A/S67D) (lanes 2 to 4). The membrane was incubated with anti-GFP (top panel) and anti-NSP5 (bottom panel). The arrows indicate the expected migration position for NSP5-CFP. The protein molecular markers are shown. Plots for the interphase stages (%) (F) and relative (S+G2)/G1 ratio (G) of MA104-Fucci cells transfected with CFP or NSP5-CFP wt, S67A or S67D. Data were acquired at 24 hpt. The relative (S+G2)/G1 ratio was calculated using CFP only cells as value of 1. Data are represented as the mean ± SEM (t-test, ** p<0.01, *** p<0.001, n = 3).
(TIF)

S1 Table List of antibodies used in this study. (DOCX)

S2 Table Primers used for plasmid construction. (DOCX)
