# Supplementary figures and images for: Rotavirus replication is correlated with S/G2 interphase arrest of the host cell cycle
Source: PLoS One. 2017 Jun 16;12(6):e0179607. doi: 10.1371/journal.pone.0179607 (PMC5473577; doi:10.1371/journal.pone.0179607)

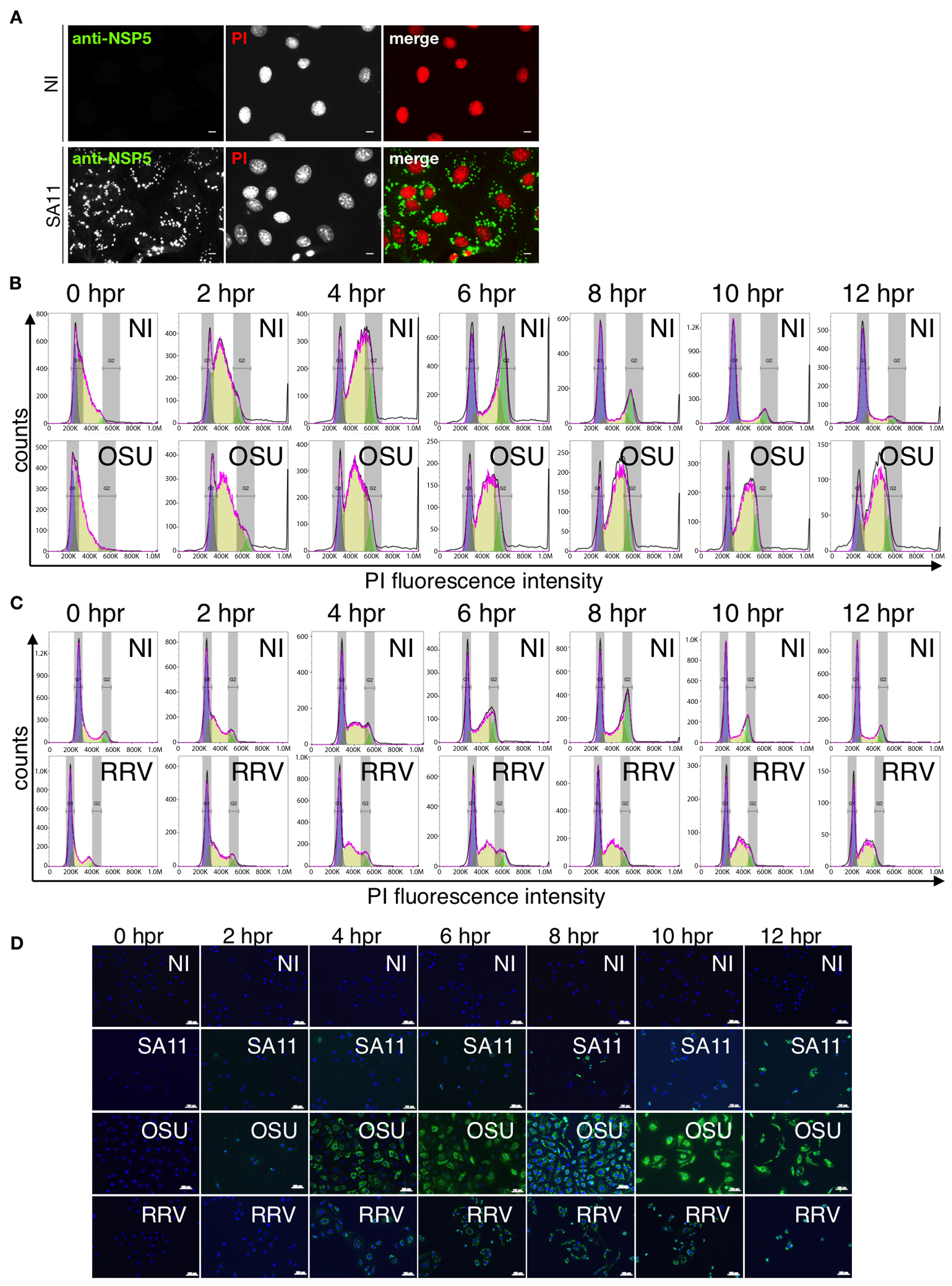

Supplement: S1 Fig — (A) Immunofluorescence of non-infected (NI) and SA11-infected MA104 cells at 6 hpi. Cells were ethanol fixed for 3 min at -20°C and immunostained for viroplasms with a specific anti-NSP5 antibody (green) and nuclei stained with PI (red). Scale bar is 10μm. Representative flow cytometry histograms of synchronized MA104 cells NI and infected [MOI, 25 VFU/cell] with porcine OSU (B) or simian RRV (C) strains acquired at 0, 2, 4, 6, 8, 10 and 12 hpr from thymidine. (D) Immunofluorescence for viral infection of cell examined for DNA content. Each histogram overlays the DNA content by Watson mathematical model where purple, yellow and green areas under the curve correspond to the percentage values of G1, S and G2 phases, respectively. Cells were immunostained for viroplasms detection (anti-NSP5, green) and stained for nuclei (DAPI, blue). Scale bar is 100 μm. (TIF) [file pone.0179607.s001.tif]

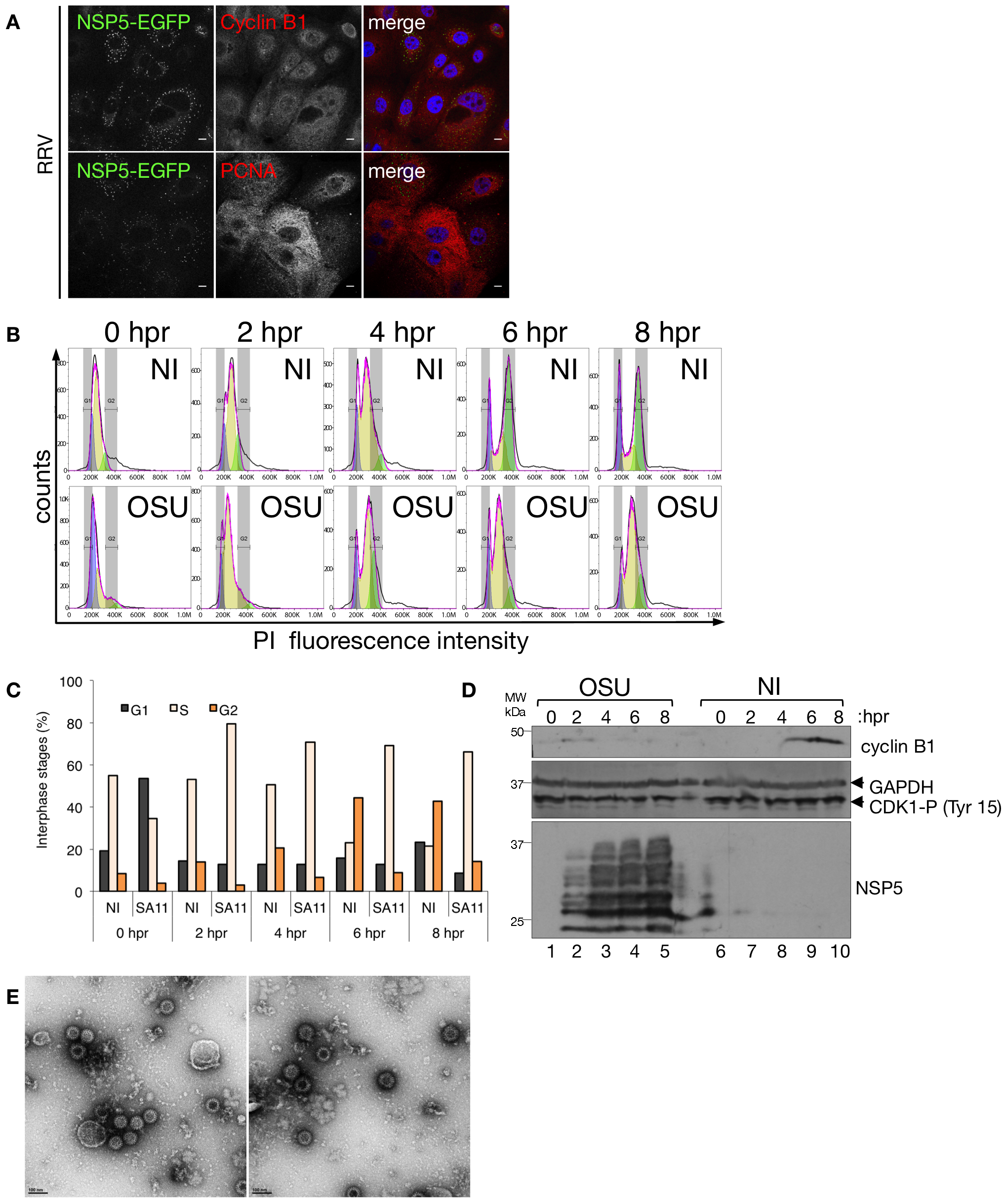

Supplement: S2 Fig — (A) Immunofluorescence of RRV-infected [MOI, 25 VFU/cell] synchronized NSP5-EGFP/MA104 cells at 6 hpr. Cells were fixed in paraformaldehyde and immunostained for cyclin B1 (mouse mAb anti-cyclin B1, red) or PCNA (mouse mAb anti-PCNA, red), viroplasms detected with NSP5-EGFP (green) and nuclei stained with DAPI (blue). The merged image is presented in the right column. Scale bar is 10μm. (B) Flow cytometer histograms of synchronized Caco-2 cells (Human colon adenocarcinoma cells) infected with porcine OSU strain [MOI, 25 VFU/cell] and analyzed at 0, 2, 4, 6 and 8 hpr from thymidine. Each histogram overlays the DNA content by DJF mathematical model where purple, yellow and green areas under the curve correspond to the values of G1, S and G2 phases, respectively. (C) Plot showing the percentage of the interphase stages (G1, S, and G2) from synchronized non-infected (NI) and OSU-infected Caco-2 cells at the indicated times post-release from thymidine. (D) Immunoblotting of cell lysates from OSU-infected (lanes 1 to 5) and non-infected (lanes 6 to10) synchronized Caco-2 cells. The cells were harvested at 0, 2, 4, 6 and 8 hpr. Cyclin B1, cdc2-P (Tyr 15) and NSP5 were detected using specific antibodies. GAPDH used as loading control. The molecular weight markers are indicated. (E) Images of electron microscopy of negatively stained OSU-TLPs after inactivation with UV-psoralen (UV/AMT). Scale bar is 100 nm. (TIF) [file pone.0179607.s002.tif]

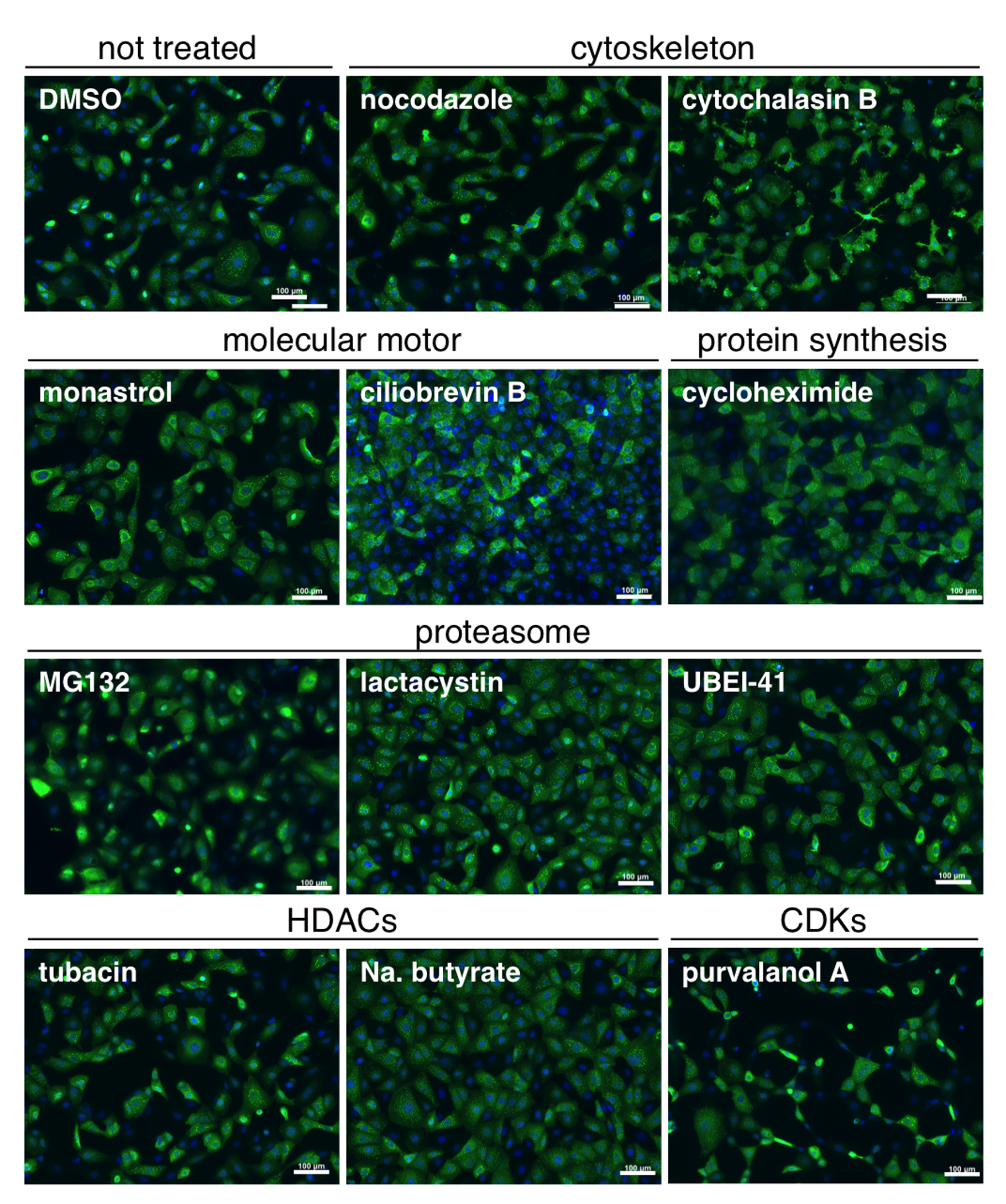

Supplement: S3 Fig — MA104 cells were infected with OSU [MOI, 25 VFU/cell] and treated at 30 min post-infection with the indicated drugs. At 8 hpi, cells were fixed, immunostained for viroplasms detection (anti-NSP5, green) and stained for nuclei (DAPI, blue). Scale bar is 100 μm. The concentration of the drugs used was: 10μM nocodazole, 10 μM cytochalasin B, 10μM monastrol, 50μM ciliobrevin D, 100μg/ml cycloheximide, 10μM MG132, 10μM lactacystin, 10μM UBEI-41, 10μM tubacin, 5mM Na-butyrate and 10μM purvalanol A. The tested drugs, at the indicated incubation time and concentration, do not induced detectable cytotoxic effect. (TIF) [file pone.0179607.s003.tif]

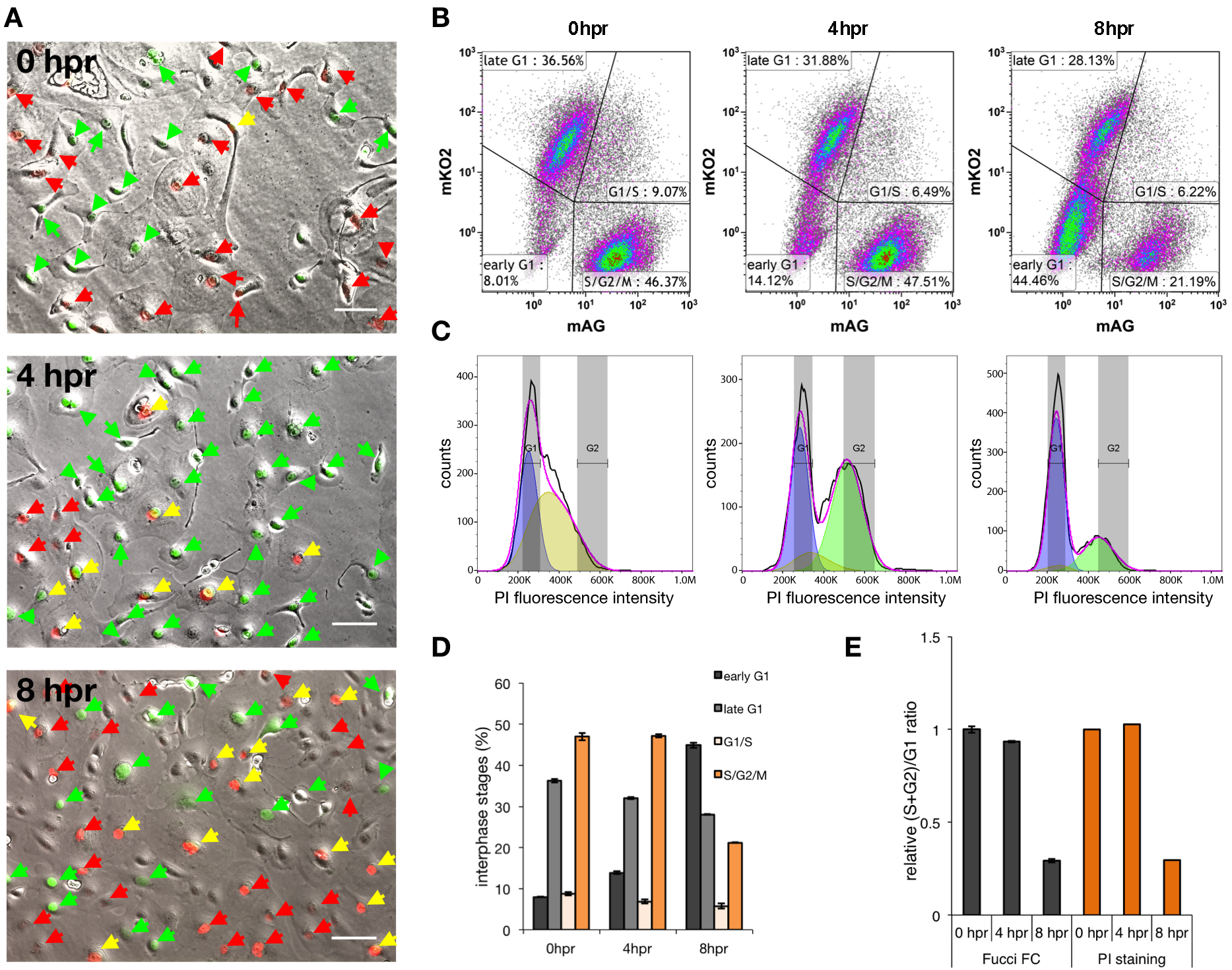

Supplement: S4 Fig — Characterization of synchronized MA104-Fucci cells at 0, 4 and 8 hpr from thymidine. (A) Fluorescence microscopy. Each image corresponds to the fluorescence merge from Ctd1-mKO2 (red), Geminin-mAG (green) and a bright field. The red, yellow and green arrowheads indicate the cells in early/late G1, G1/S and S/G2/M phases, respectively. Scale bar is 100μm. (B) Density plots.The cells were discriminated by its Ctd1-mKO2 (red) and Geminin-mAG (green) fluorescence intensities and gated as early G1, late G1, G1/S and S/G2/M. (C) DNA content histograms determined by PI fluorescence intensity. The data were obtained using DJF model where purple, yellow and green areas under the curve correspond to the values of G1, S and G2 phases, respectively. G1 and G2 phases were constrained. (D) Interphase stages plot (early/late G1, G1/S and S/G2/M). (E) Comparison plot of relative (S+G2)/G1 ratio obtained from flow cytometry of fluorescence intensities of Ctd1-mKO2 and Geminin-mAG (gray bars) or DNA content of PI fluorescence intensity (orange bars). The relative (S+G2)/G1 ratio was calculated considering NI cells at 0 hpr as a value of 1. Data represented the mean ± SEM, from three independent experiments. (TIF) [file pone.0179607.s004.tif]

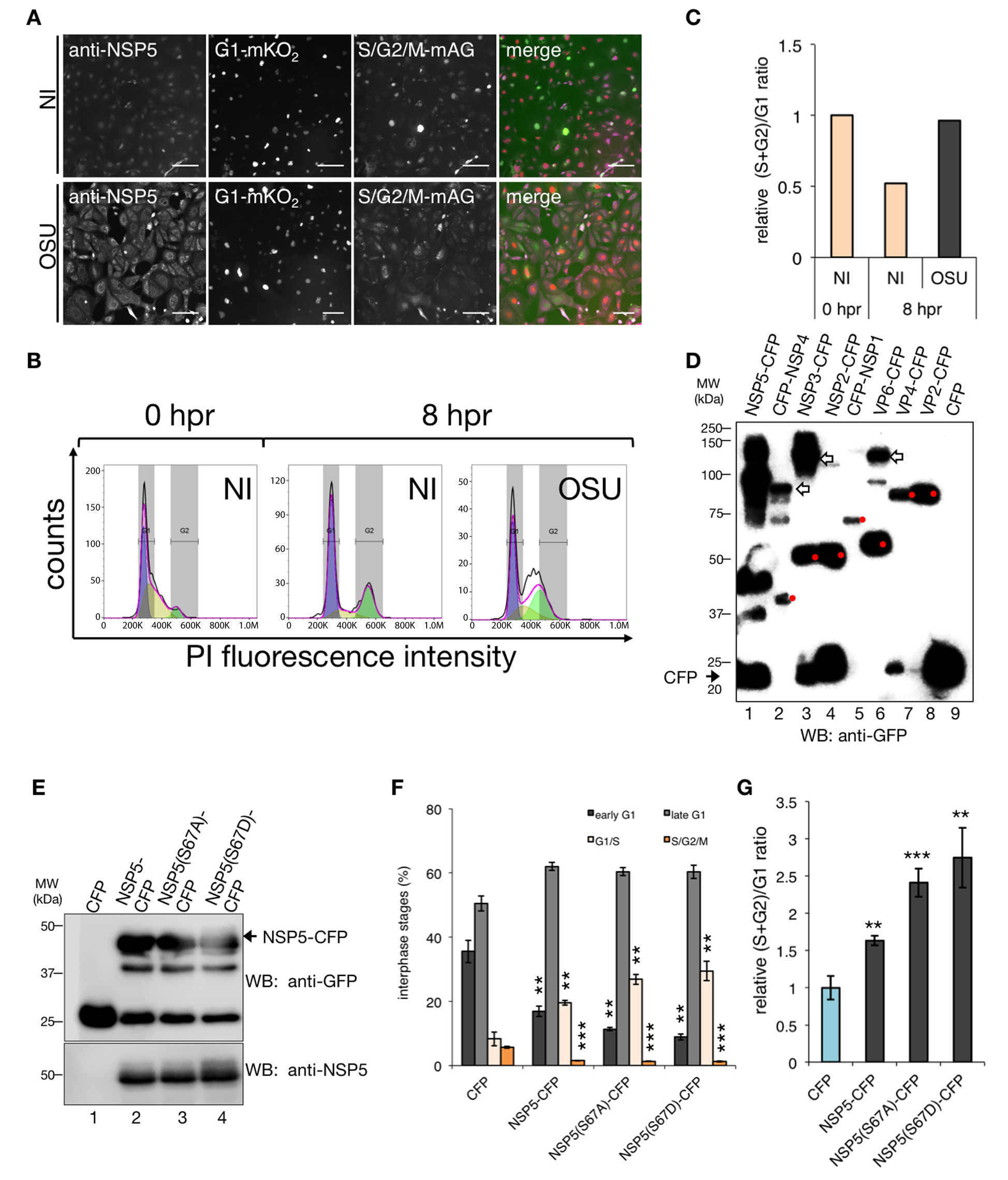

Supplement: S5 Fig — Characterization of NI and OSU-infected [MOI, 25 VFU/cell] synchronized MA104-Fucci cells after 0 and 8 hpr from thymidine. (A) Immunofluorescence of NI (upper row) and OSU-infected (lower row) synchronized MA104-Fucci cells. Cells were immunostained at 8 hpr for viroplasms detection (anti-NSP5, magenta, left column). Fucci sensors G1-mKO2 (red) and S/G2/M-mAG (green) are indicated (middle columns). A merged image is shown in the right column. Scale bar is 100 μm. (B) DNA content determined by PI fluorescence intensity. The cell cycle was calculated using DJF mathematical model where purple, yellow and green areas under the curve are the percentage values of G1, S and G2 phases, respectively. G1 and G2 phases were constrained. (C) The plot of relative (S+G2)/G1 ratio 0 and 8 hpr. (D) Immunoblotting of cell lysates of MA104 expressing RV proteins fused to CFP. The cells were transfected in the presence of VVT7.3 and lysed at 16 hpt. The membrane was incubated with anti-GFP antibody followed by the corresponding secondary antibody conjugated to peroxidase. The red dot and white arrows indicate the expected migration position for monomeric and oligomeric forms of each of the fusion proteins. The protein molecular markers are shown. The CFP tag was added at the N- or C-terminus of each RV protein as described elsewhere. Each RV protein fused to CFP was tested for functional expression by fluorescence microscopy at 24 hpt (data not shown). (E) Immunoblotting of cell lysates of MA104 expressing CFP (lane 1), NSP5-CFP wt or NSP5-CFP carrying the point mutations S67A and S67D (S63, 65A/S67D) (lanes 2 to 4). The membrane was incubated with anti-GFP (top panel) and anti-NSP5 (bottom panel). The arrows indicate the expected migration position for NSP5-CFP. The protein molecular markers are shown. Plots for the interphase stages (%) (F) and relative (S+G2)/G1 ratio (G) of MA104-Fucci cells transfected with CFP or NSP5-CFP wt, S67A or S67D. Data were acquired at 24 hpt. The rela [file pone.0179607.s005.tif]
