# Supplementary material for: Rotavirus replication is correlated with S/G2 interphase arrest of the host cell cycle
Source: PLoS One. 2017 Jun 16;12(6):e0179607. doi: 10.1371/journal.pone.0179607 (PMC5473577; doi:10.1371/journal.pone.0179607)
Supplement: S1 Table — (DOCX) [file pone.0179607.s006.docx]

**S1 Table. List of antibodies used in this study.**

| **Antibody** | **Reference/Source** |
| --- | --- |
| Mouse mAb anti-NSP5 | Petris et al., 2014 [76] |
| Guinea pig polyclonal anti-NSP5 serum | Eichwald et al. 2004 [16] |
| Guinea pig polyclonal anti-NSP2 serum | Eichwald et al., 2004[16] |
| Guinea pig polyclonal anti-RV serum | Eichwald et al., 2012 [18] |
| Rabbit anti-NSP3 | A gift from Dr. S. López |
| Mouse mAb anti-Eg5 (clone 10C7/Eg5) | Biolegend^®^ |
| Rabbit anti-Eg5 phospho Thr927 (poly6205) | Biolegend^®^ |
| Mouse mAb anti-cyclin B1 (clone V152) | Biolegend^®^ |
| Rabbit polyclonal anti-phospho-cdc2 (tyr15) | Cell Signalling Technology^®^. |
| Mouse polyclonal anti-cdc-2 | Cell Signalling Technology^®^. |
| Mouse mAb anti-human cyclin A (clone 25/cyclin) | BD, Biosciences. |
| Mouse mAb anti-hEg5 (clone 20/EG5) | BD, Biosciences. |
| Mouse mAb anti-GFP (B-2) | Santa Cruz Biotechnology |
| Mouse mAb anti-GAPDH (clone GAPDH-71.1) | Sigma-Aldrich |
| Goat anti-mouse IgG (H+L) Alexa 488 | Molecular Probes, Invitrogen, USA. |
| Rabbit anti-mouse F (ab’)2 fragments Alexa 594 | Molecular Probes, Invitrogen, USA. |
| Goat anti-mouse IgG Alexa 647 | Molecular Probes, Invitrogen, USA. |
| Goat anti-guinea pig IgG (H+L) Alexa 488 | Molecular Probes, Invitrogen, USA. |
| Goat polyclonal anti-guinea pig IgG conjugated to rhodamine | KPL, USA. |
| Goat polyclonal anti-mouse IgG (Fab)’-peroxidase | Sigma-Aldrich |
| Rabbit polyclonal anti-guinea pig Ig peroxidase | Dako Cytomation, Denmark. |
| Donkey anti-rabbit IgG-IRDye®800CW | LI-COR, USA. |
| Donkey anti-mouse IgG IRDye®680RD | LI-COR, USA. |

^a^mAb, monoclonal antibody

^b^IgG, Immunoglobulin G
