# Supplementary material for: Rotavirus replication is correlated with S/G2 interphase arrest of the host cell cycle
Source: PLoS One. 2017 Jun 16;12(6):e0179607. doi: 10.1371/journal.pone.0179607 (PMC5473577; doi:10.1371/journal.pone.0179607)
Supplement: S2 Table — (DOCX) [file pone.0179607.s007.docx]

**S2 Table. Primers used for plasmid construction.**

| **Amplified segment** | **Oligonucleotide sequences** |
| --- | --- |
| Fucci-G1o | Fwd.:5’-GATCGGATCCCCACC**ATG**GTGAGTGTGATTAA-3’  Rev.:5’-GACGAATTC**TTA**GATGGTGTCCTGGTCCTGCGC-3’ |
| Fucci-S/G2/Mg | Fwd.:5’-GATCGGATCCCCACC**ATG**GTGAGCGTGATCAA-3’  Rev.:5’-GATCGAATTC**TTA**CAGCGCCTTTCTCCGTTTTTC-3 |
| CFP-N | Fwd.:5’-GATCCTCGAG**ATG**GTGAGCAAGGGCGAGGAG -3’  Rev.:5’-AGTCACGCGTCTTGTACAGCTCGTCCATGCC-3’ |
| atg-CFP | Fwd.:5’-GATCCTCGAG**ATG**GTGAGCAAGGGCGAGGAG-3’  Rev.:5’-GATCGCGGCCGC**TTA**CTTGTACAGCTCGTCCAT-3’ |
| C-CFP | Fwd.:5’-GATCACGCGTGTGAGCAAGGGCGAGGAGCTG-3’  Rev.:5’-GATCGCGGCCGC**TTA**CTTGTACAGCTCGTCCCAT-3’ |
| VP6 | Fwd.:5’-GATCACGCGTG**ATG**TGCTGTACTCCATCTCA-3’  Rev.:5’-GATCGCGGCCGC**TCA**CTTTACCAGCATGCTTCT-3’ |
| NSP1 | Fwd.:5’-GATCACGCGTGCTACTTTTAAAGATGCATGC-3’  Rev.:5’-GATCGCGGCCGCCAC**TTA**TCATTGTCATCTTCTGAG-3’ |
| NSP4 | Fwd.:5’-GACTACGCGTGATAAGCTTGCCGACCTC-3’  Rev.:5’-GATCGCGGCCGC**TTA**CACATGGATGCAGTCACTTCT-3’ |
| VP4 | Fwd.:5’-GATCCTCGAG**ATG**GCTTCGCTCATTTATAGA-3’  Rev.:5’-GATCACGCGTCAACCTGCATTGCATAATCAGT-3’ |
| NSP3 | Fwd.:5’-GATCCTCGAG**ATG**CTCAAGATGGAGTCTACG-3  Rev.:5’-GATCACGCGTTTCGCAACCAAATGAATATTG-3’ |
| NSP2 | Fwd.:5’-GATCGAATTCATGGCTGAGCTAGCTTGCTTT-3’  Rev.:5’-GATCACGCGTAACGCCAACTTGAGAAACTTC-3’ |
| VP2 | Fwd.:5’-GATCGAATTC**ATG**GCGTATCGAAAACGTGGA-3’  Rev.:5’-GATCCACGCGTCAGTTCGTTCATGATGCGCAT-3’ |
| NSP5wt  NSP5 (S67A)  NSP5(S67D) | Fwd.:5’-GATCGAATTC**ATG**TCTCTCAGTATTGACGTG-3’  Rev.:5’-GATCACGCGTCAAATCTTCAATCAATTGCAT-3’ |
| gs5 | Fwd.:5’-AGGTACCTAATACGACTCACTATAGGCTTTTTTTTGAAAAGTC-3’  Rev.:5’-ACCGCGGTCACAGTATTTTGCCAGCTA-3’ |

^a^ Restriction enzyme recognition sites are underlined.

^b^ Initiation and stop codons are labeled in bold.
